# DNA hybridization kinetics: zippering, internal displacement and sequence dependence

**DOI:** 10.1093/nar/gkt687

**Published:** 2013-08-08

**Authors:** Thomas E. Ouldridge, Petr Šulc, Flavio Romano, Jonathan P. K. Doye, Ard A. Louis

**Affiliations:** ^1^Rudolf Peierls Centre for Theoretical Physics, Department of Physics, University of Oxford, 1 Keble Road, OX1 3NP, Oxford, UK and ^2^Physical & Theoretical Chemistry Laboratory, Department of Chemistry, University of Oxford, South Parks Road, OX1 3QZ, Oxford, UK

## Abstract

Although the thermodynamics of DNA hybridization is generally well established, the kinetics of this classic transition is less well understood. Providing such understanding has new urgency because DNA nanotechnology often depends critically on binding rates. Here, we explore DNA oligomer hybridization kinetics using a coarse-grained model. Strand association proceeds through a complex set of intermediate states, with successful binding events initiated by a few metastable base-pairing interactions, followed by zippering of the remaining bonds. But despite reasonably strong interstrand interactions, initial contacts frequently dissociate because typical configurations in which they form differ from typical states of similar enthalpy in the double-stranded equilibrium ensemble. Initial contacts must be stabilized by two or three base pairs before full zippering is likely, resulting in negative effective activation enthalpies. Non-Arrhenius behavior arises because the number of base pairs required for nucleation increases with temperature. In addition, we observe two alternative pathways—pseudoknot and inchworm internal displacement—through which misaligned duplexes can rearrange to form duplexes. These pathways accelerate hybridization. Our results explain why experimentally observed association rates of GC-rich oligomers are higher than rates of AT- rich equivalents, and more generally demonstrate how association rates can be modulated by sequence choice.

## INTRODUCTION

DNA is central to biology and has become a key ingredient in nanotechnology. Single strands of DNA have a sugar-phosphate backbone with bases (adenosine, thymine, guanine or cytosine—hereafter referred to as A, T, G and C) attached at regular intervals. Watson and Crick (1) showed that hydrogen bonding between A-T and G-C base pairs and stacking interactions between adjacent bases result in helical duplexes when two sequences are complementary. This rule has been used to design structures (2), machines (3) and computational circuits (4) that operate in parallel on a nanometer length scale. In many of these systems, assembly or operational dynamics is primarily driven by the association, or hybridization, of short strands of DNA (oligomers) to form duplexes of order ten base pairs. Understanding the details of oligomer association kinetics, and knowledge of how to accelerate or suppress reaction rates, is therefore essential if DNA nanotechnology is to fulfil its promise.

The thermodynamics of DNA duplex formation is dominated by states involving either strongly bound duplexes or widely separated strands. Therefore, it is well characterized (5,6) and can be described by all-or-nothing (two-state) models (5). By contrast, hybridization kinetics depends on the rarely visited intermediate states that lie between these two limits and is therefore much harder to understand. In principle, the ensemble of transition pathways may be complex, leading to rich and subtle behaviour. Bimolecular association rate constants of 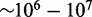 M^−^^1^s^−^^1^ have been measured at approximately room temperature and at high salt concentrations ([Na^+^] ∼1 M or [Mg^2+^] ∼ 0.01 M) for DNA (7–9) and RNA (10–13). There is agreement that dissociation rates increase exponentially with temperature (8,10–12,14), but authors have reported association rates that increase (8,12), decrease (10,11) and behave non-monotonically (14) with temperature. To our knowledge, there have been no systematic studies of the consequences of DNA sequence for hybridization rates.

Theoretical models at the level of secondary structure (the degree of base pairing) have been proposed to explain experiments where association rates decrease with temperature (10,11,14). These models posit that strands initially held together by short duplex sections tend to dissociate rather than fully hybridizing, either because they melt extremely quickly (10,11) or owing to a thermodynamic barrier to full hybridization (14). However, hairpins with stems as short as three base pairs are thermodynamically stable (7), and no detailed description of a barrier to completing hybridization has been proposed.

Computer modelling can shed light on the details of hybridization kinetics. Ideally, simulations would use atomistic potentials such as AMBER (15) for maximal resolution, but the long time scales involved prevent exhaustive studies of such systems. To explore reaction pathways, coarse-grained models are needed. These must be efficient enough to access the critical time-scales but detailed enough to represent key features of the 3D structure, the mechanical properties and the thermodynamics of both single- *and* double-stranded DNA. A number of models have been proposed, but most are either ‘ladder’ models that do not capture structural and mechanical properties of DNA (16–20) or have not been carefully parameterized to DNA thermodynamics (21–25). Hybridization kinetics have been studied in a detailed model known as 3SPN.1 (26,27). The authors identified initial binding and then ‘slithering’ of strands past each other as a mechanism of duplex formation. However, single strands in 3SPN.1 are overly stiff and have structural and mechanical properties that are similar to the duplex state; therefore, association necessarily involves two pre-formed helices coming into contact. It is unclear whether the same pathway will be observed for a model with a more realistic description of the single-stranded state.

Here, we apply a recently developed coarse-grained model, ‘oxDNA’ (28–30), to the association of DNA oligomers. The model incorporates the average structural, mechanical and thermodynamic properties of both single- and double-stranded DNA. Duplexes are stiff helices, but single strands can unstack, allowing them to adopt non-helical structures, reproducing their relative flexibility. Such flexibility allows oxDNA to capture properties such as the formation of hairpins (28) and the force-extension properties of single strands (31), and we expect the flexibility of single strands to be a critical factor in hybridization. The robustness of oxDNA has been established by studying a range of phenomena that were not used for the initial parameterization. The formation of metastable kissing hairpins (32), cruciform structures under torsion (33) and liquid crystals at high density (34) are all reproduced in a physically reasonable way. Three dynamic DNA-based nanodevices have also been simulated (35,36, P. Šulc, T. E. Ouldridge, F. Romano, J. P. K. Doye, and A. A. Louis, submitted for publication.). These calculations not only reproduced the designed behaviour but also identified key subtleties arising from an interplay of structural, mechanical and thermodynamic factors. oxDNA undergoes an overstretching transition, with the critical force in good agreement with experiment (31). Most importantly, oxDNA quantitatively reproduces the 10^6.5^-fold acceleration of the toehold-mediated strand displacement rate with increasing toehold length (N. Srinivas, T. E. Ouldridge, P. Šulc, J. Schaeffer, B. Yurke, A. A. Louis, J. P. K. Doye, and E. Winfree, submitted for publication) found by Zhang and Winfree (9). As binding to the toehold involves the same self-assembly processes as in hybridization, our success gives us confidence in using oxDNA to study oligomer association in detail.

We proceed as follows. After briefly presenting the model and simulation techniques, we study hybridization processes for sequences designed to limit misbonding. Hybridization involves a zipper-like mechanism, and reaction rates are suppressed at increased temperature due to the instability of initial contacts. We then consider repetitive sequences, finding that alternative pathways to duplex formation, which we name ‘inchworm’ and ‘pseudoknot’ internal displacement, can significantly accelerate association. Finally, we demonstrate that the instability of initial contacts and alternative pathways to assembly can lead to sequence-dependent hybridization rates, in agreement with some recent experiments (9).

## MATERIALS AND METHODS

### A coarse-grained model

**Figure 1.**
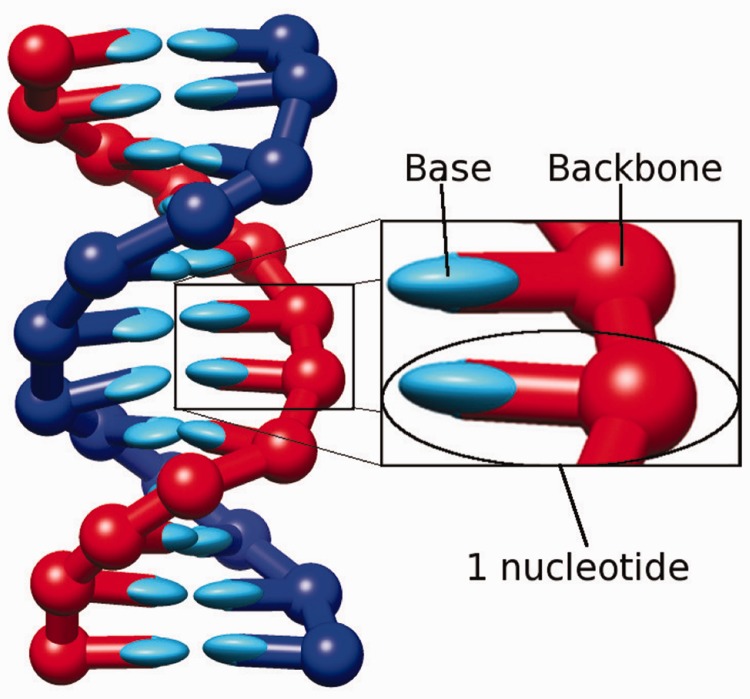
A 12-bp duplex in oxDNA. Two rigid nucleotides are shown in the enlarged box, with backbone and base highlighted. Bases are represented as non-spherical ellipsoids as interaction potentials are explicitly dependent on relative nucleotide orientations.

oxDNA is detailed in (28–30), and in Supplementary Section S1. The version used for the majority of this work is given in (29), and code implementing it is available for download (37). A model strand is a chain of rigid bodies, each one representing a nucleotide. Nucleotides have one interaction site for the backbone and two for the base. The potential energy of the system includes terms for backbone connectivity, base-pairing, stacking (including cross-stacking interactions between strands) and excluded volume interactions (illustrated in Supplementary Figure S1). The combined result of the interactions is that complementary strands tend to form helical duplexes, as shown in Figure 1.

Base-pairing interactions are only included between complementary pairs A-T and G-C to reproduce Watson–Crick specificity. For much of this work, we use a parameterization with no further sequence dependence (29) to highlight generic properties that can be obscured by sequence-dependent effects. For sequence-dependent results, we use a parameterization in which hydrogen-bonding and nearest-neighbour stacking strengths depend on the identity of the bases (30). Both parameterizations were fitted to oligonucleotide melting temperatures predicted by SantaLucia’s nearest-neighbour model (5), as well as the structural and mechanical properties of double- and single-stranded DNA. The model was fitted to experiments performed at 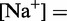 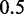 M, a high salt concentration at which the strength of screening justifies incorporating electrostatic repulsion into a short-ranged excluded volume.

### Simulation techniques

The majority of simulations in this work were performed using a Langevin Dynamics (LD) algorithm (38). LD represents an implicit solvent by augmenting the Newtonian equations of motion with drag and random noise forces. Simulated particles then undergo diffusive motion, and the whole system samples the canonical ensemble if the relative sizes of the drag and noise forces are chosen appropriately (38). Details of our implementation are given in Supplementary Section S2A. As is common in simulations of coarse-grained models, we use a higher diffusion coefficient than for physical DNA. The freedom to accelerate diffusion is an advantage of coarse-grained models, which also tend to increase the speed with which processes occur by smoothing energy landscapes on a microscopic scale (39). As a result, they can be used to study even more complex systems than would otherwise be expected. We will generally focus on relative rates in this work, as for qualitatively similar processes, our simplified model and kinetics will have a smaller effect on the ratios of rates than on their absolute values.

To check that the results reported here are not overly sensitive to the details of the simulation method and choice of friction constants, hybridization of non-repetitive duplexes was also simulated with diffusion coefficients reduced by a factor of 10. The results (Supplementary Table S3, discussed in Supplementary Section S2A) are qualitatively similar, except of course for an overall drop in the reaction rate with slower dynamics. For the sequence-dependent results at the end of this work, an alternative Brownian thermostat (40) was used as detailed in Supplementary Section S2B. As we will show, the Brownian algorithm (with a larger diffusion coefficient than the LD approach) produces behaviour consistent with the predictions of the LD thermostat, further evidence that the qualitative results of this work are not sensitive to the simulation method.

To obtain good statistics for reaction transitions, which are dominated by rare events, we used Forward Flux Sampling (FFS) (41,42). This technique facilitates sampling of a complex transition path ensemble by splitting a rare event into several stages (the crossing of intermediate interfaces) as depicted in Supplementary Figure S2. Details of the application of FFS in this work are provided in Supplementary Sections S2A.i and S3A–D, with order parameters and results tabulated in Supplementary Tables S1–S11. Finally, simulations performed to measure equilibrium averages, rather than dynamics, were performed with an efficient cluster-move Monte Carlo algorithm (43), with the addition of umbrella sampling (44). Details are given in Supplementary Sections S2C and S3E, and the umbrella potentials used in Supplementary Table S12.

## RESULTS AND DISCUSSION

### Hybridization of non-repetitive sequences

We first consider the hybridization of 14-base strands deliberately designed to limit non-intended base pairing. The sequences of the two strands are as follows:

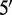
-TAT CTG GCT TGT CG-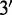,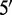-CGA CAA GCC AGA TA-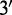.

Simulations were run at a range of temperatures, from 300 K to 340.9 K, the latter being approximately the melting temperature of the strands at the concentration used. Details of the simulations are provided in Supplementary Section S3.A. Additional simulations at 300 K were performed in which only native (those expected in the full 14-base pair duplex) base pairs were assigned a non-zero hydrogen-bonding energy, to determine the effect of non-native base pairs.

**Figure 2.**
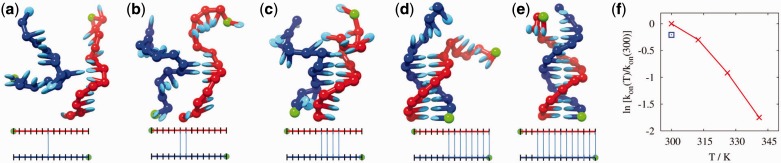
(**a–e**) Stages of DNA hybridization taken from a typical single trajectory. Green spheres indicate the 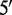 end of each strand. Schematic diagrams underneath indicate the base pairs present in the system. (**f**) Hybridization rates 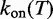 of non-repetitive duplexes as a function of temperature *T*, relative to 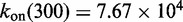 s^−1^, shown as red crosses connected by a solid line. Also shown (blue square) is the result for duplex formation at 300 K when misaligned bonds are forbidden.

Qualitatively, successful binding events involve the diffusion of the strands into contact, which can lead to favourable base pairing and cross-stacking interactions between strands. Generally, a strong base pair must form quickly or these contacts will dissociate. In successful binding events, more base pairs subsequently form before this base pair breaks, a process postulated elsewhere (10,11,45,46) and known as ‘zippering’. A typical pathway is illustrated in Figure 2a–e. In some cases, initial base pairs are non-native, with native base pairs replacing them later. From Figure 2, it is clear that zippering involves relatively unstructured single strands coming together to form base pairs in stages. Initial contacts can form between any bases but have a bias towards those at the end of the strands, although initial contacts in the centre are more likely to proceed to the full duplex once formed (as shown in Supplementary Figure S4).

**Figure 3.**
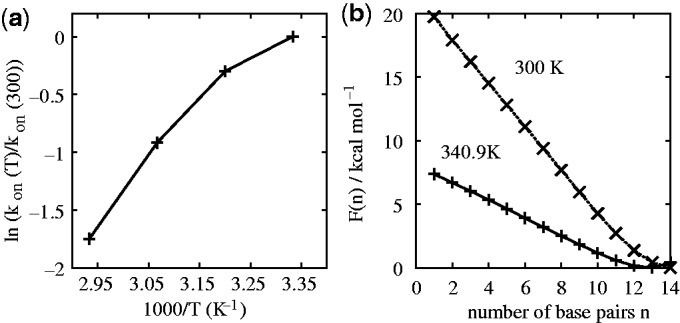
(**a**) ‘Arrhenius plot’ of the dependence of hybridization rate on temperature, with 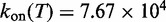 s^−1^ being the rate at 300 K. The Arrhenius model with a constant activation enthalpy predicts a straight line on such a plot. (**b**) Free-energy profile of the duplex state for a 14-base pair duplex with interactions exclusively between native base pairs, measured at 300 K and 340.9 K, obtained as outlined in Supplementary Section S3E. The free energy *F*(*n*) is a measure of the equilibrium probability of having *n* base pairs, *P*(*n*): 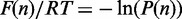. The arbitrary offsets of the free energies are chosen so that the minima of both curves have a value of zero. These simulations did not sample the unbound state, and hence they are concentration independent. We do not show the single-stranded state (0 base pairs) because this is concentration dependent, in contrast to the rest of the free-energy profile. For examples with this state included, see, for example, (28–30).

Figure 2f shows the binding rate as a function of temperature. It is evident that the binding rate decreases with increasing temperature. In the commonly used Arrhenius model of reaction kinetics, the association rate 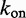 depends on the temperature *T* as 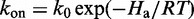, where 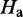 is a constant activation enthalpy, and *k*_0_ is a constant rate. A system with a single well-defined transition state would follow this prediction. A decrease in 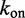 with *T* suggests a transition state with a negative enthalpy with respect to the unbound state. Overall, however, the Arrhenius model is a poor fit to our results, as can be seen in Figure 3a. The apparent activation enthalpy, which can be inferred from the slope 
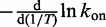
, becomes more negative with temperature, with our model showing an apparent 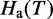 ranging from around −4.5 kcal mol^−^^1^ to approximately −12.5 kcal mol^−^^1^, as *T* rises from 300 K to 340.9 K. These values are similar to those measured for short RNA oligomers, which range from −5 kcal mol^−^^1^ (10) to −9 to −18 kcal mol^−^^1^ (11).

In contrast to association, we expect a relatively large positive activation enthalpy of dissociation because breaking a fully formed duplex involves disrupting many enthalpically favoured bonds. This explains why experimental measurements find dissociation rates 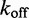 that increase exponentially with increasing temperature (8,10–12,14). Indeed, from calculations of the equilibrium constant 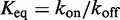 (28), we find that 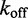 changes by 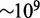 over the range 300–340.9 K. Here, we focus on the more subtle behaviour of the association rate, which is especially relevant for non-equilibrium processes in DNA nanotechnology.

FFS allows us to sample from the ensemble of transition pathways. In particular, it provides us with an ensemble of states at several stages of the process (each FFS interface), and a success probability of duplex formation when each of these interfaces is reached. In our simulations of association, the penultimate interface of the FFS simulations is of most interest: configurations found here have two base pairs with significant interactions (see Supplementary Section S3A for detailed definitions).

At 300 K, states at this penultimate FFS interface have a 33% probability of reaching the full duplex, whereas at 340.9 K, this success rate drops to just 8% (see Supplementary Table S13 for other temperatures). Even for systems with only native base pairing allowed, states with two relatively well-formed base pairs still only progress to the full duplex in 65% of cases at 300 K. The fact that states with some base pairing can fail to form a duplex explains the negative activation enthalpy: our effective ‘transition state’ is enthalpically stabilized by base pairing. Further, the typical number of base pairs in this ‘transition state’ increases with temperature, as more base pairing is required to make duplex formation probable. The reasons for the temperature dependence include the following: (i) the state with two base pairs itself becomes less stable and (ii) new bonds are less likely to form because (a) strands become more unstructured and (b) forming new base pairs generates a smaller free-energy gain. As a result, the activation enthalpy becomes more negative with temperature, and there is no single transition state with well-defined properties, explaining the non-Arrhenius behaviour.

**Figure 4.**
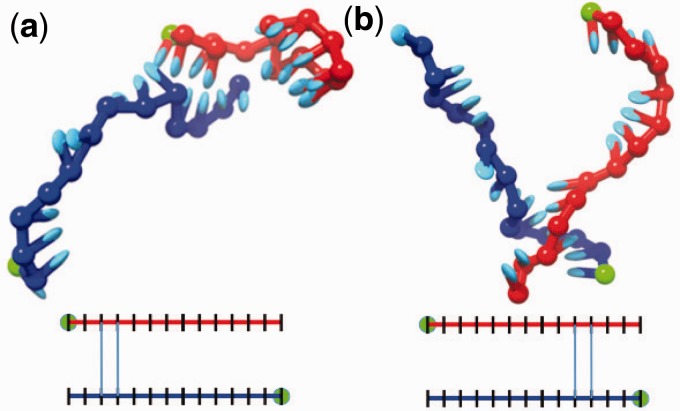
Two configurations with two base pairs, and an interstrand enthalpy of 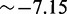 kcal mol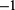. The configuration in (**a**) is taken from a simulation of association and that in (**b**) from an equilibrium simulation of the system.

Simple thermodynamic considerations at the level of secondary structure do not explain why many initial contacts fail to completely hybridize. For example, free-energy profiles of the duplex states, obtained from equilibrium simulations outlined in Supplementary Section S3E and plotted in Figure 3b, suggest that adding a single base pair reduces the free-energy of the system by 0.6 kcal mol^−^^1^ or 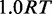 at 340.9 K, and 1.7 kcal mol^−^^1^ or 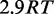 at 300 K. This argument suggests that the process should be favourable once the first base pair has formed. To understand why this reasoning fails, we compare configurations obtained from hybridization simulations to configurations with the same degree of base pairing taken from equilibrium duplex simulations. In Figure 4a and b, we show two configurations with the same base pairing and overall interstrand enthalpy, with panel (a) obtained from a simulation of association initiated in the unbound state and panel (b) obtained from equilibrium simulations of the bound state. Clearly, the latter has a much more favourable spatial conformation for full duplex formation, as less rearrangement is required. A thorough analysis (the methodology is detailed in Supplementary Section S3E) confirms that states found at the penultimate FFS interface (which have two clearly formed base pairs) are on average different from those with the same degree of base pairing found in equilibrium simulations, and clearly less conducive to full duplex formation (see Supplementary Table S14). For example, we find that bases tend to be further away from their native partners in states drawn from the kinetic ensemble, with an average separation of 2.84 nm rather than 2.10 nm. We emphasize that this argument does not violate microscopic reversibility: we find that the states visited during duplex formation (and, we infer, during dissociation as well) with a certain number of base pairs have properties that are not typical of states with the same degree of base pairing drawn from an equilibrium ensemble.

The aforementioned argument requires that the breaking of the initial contacts can occur faster than strands equilibrate in the configuration space available given the existence of those contacts. This is plausible because the single strands are disordered. Thus, the system appears non-Markovian when analysed only in terms of secondary structure: a given state has memory of whether it is accessed during assembly transitions or accessed from the bound ensemble. We stress that it is the realistic 3D structure of single and double strands that allow oxDNA to capture such non-equilibrium effects, which are absent in typical models that are constructed at the secondary structure level (47,48). We note that this argument is sensitive to the relative rates of base pairing, internal equilibration of strands and centre of mass diffusion, which may vary from those in the model: if base pairing timescales were relatively much slower, the system would not appear non-Markovian. The effect in our model is clear, however, and it seems intuitively reasonable that the time scale of base pair disruption should be short compared with the timescale of structural equilibration of an oligomer, which involves many degrees of freedom and interactions between nucleotides.

### Hybridization of repetitive sequences

Having studied strands in which interactions involving non-aligned strands are minimal, we now consider the limit of repetitive sequences that can form many misaligned structures (but no intrastrand hairpins). The sequences of the two strands are as follows:

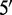
-ACA CAC ACA CAC AC-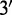,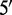-GTG TGT GTG TGT GT-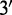.

**Figure 5.**
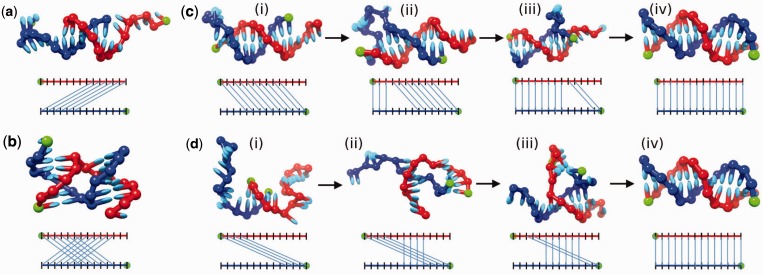
(**a**) Misaligned bonding in register −8. (**b**) Pseudoknot bonding, in registers 6 and −6. Examples of internal displacement through (**c**) the inchworm mechanism and (**d**) the pseudoknot mechanism. (c.i) The system is initially bound through 10 base pairs in register 4. (c.ii) Owing to thermal fluctuations, three base pairs from register 0 form at the expense of three base pairs from register 4, resulting in a ‘bulge’. (c.iii) More base pairs in register 0 form at the expense of register 4. The bulge is passed down the helix. (c.iv) Eventually, the final base pair in register 4 breaks and register 0 is able to form all its possible base pairs. (d.i) A duplex initially in register 10. (d.ii) Additional base pairs (from the correctly aligned register 0) form. (d.iii) Further base pairs form in register 0 at the expense of register 10. (d.iv) Eventually, the final base pair in register 10 breaks and a fully bonded correctly aligned duplex can form.

At 300 K, we find a number of metastable structures in addition to the fully bound duplex. These structures involve misaligned duplexes, which we label by their ‘register’. A register of *r* corresponds to bases pairing with a partner offset by *r* bases in the 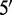 direction from their native partner. The simulations indicate that two classes of structures can be relatively long-lived:

- Purely misaligned structures with the maximum number of base pairs given their register. A configuration with the maximal bonding for register −8 is illustrated in Figure 5a.
- ‘Pseudoknot’ structures (6), characterized by two registers *r*_1_ and *r*_2_. If we label nucleotides by their position on the strand (in the 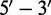 direction), then in a pseudoknot, the index of bases involved in pairing on one strand is a non-monotonic function of the index of their partner on the other strand. A typical metastable structure involving registers 6 and −6 is illustrated in Figure 5b.

These metastable structures can be relatively slow to relax into either the fully formed duplex or dissociated single strands. FFS is not efficient when intermediates with long lifetimes are present. We therefore initially measured the rates at which strands formed a misaligned structure of at least four base pairs, or a number of the more stable pseudoknot states, at 300 K. Further FFS simulations were performed to establish the eventual fate of a number of metastable states. Details are provided in Supplementary Sections S3B–D.

Strands can initially associate through zippering in a range of registers (see Supplementary Table S15 for details). The rate of formation of a given register increases with the number of base pairs in that register, owing to the number of possible initial contacts (long-lived pseudoknots typically do not form directly, but rather with one register following another as will be discussed later in the text). From this point, systems with incomplete base pairing tend to rearrange into registers with a greater degree of base pairing. We describe these rearrangement processes as ‘internal displacement’, as they involve the formation of a secondary double helix of an alternative register that competes for base pairing with the first. This is analogous to the well known ‘strand displacement’ process in which an invading strand replaces another within a duplex, except that in this case only two strands are involved (in a sense, a strand displaces itself). Two dominant rearrangement processes are observed.

- ‘Inchworm’ displacement (Figure 5c): thermal fluctuations allow base pairs from an alternative register to form. The result is a ‘bulge’ loop (5). Generally, this (unfavorable) bulge is resolved by breaking the newly formed base pairs in the alternative register. Occasionally, however, further base pairs are broken in the original register, and additional base pairs in the new register form. The bulge can thus be passed through the original duplex in an inchworm fashion, allowing the new register to displace the old.
- ‘Pseudoknot’ displacement (Figure 5d): short misaligned duplexes have two long single-stranded tails. These tails can bind, resulting in a pseudoknot. The new register can compete for base pairs with the old, potentially displacing it. Some of these pseudoknots quickly resolve into one register or the other. In other cases, such as the pseudoknot −6,6 in Figure 5b, neither arm of the pseudoknot can fully displace the other, and spontaneous melting of several base pairs in one register without them being immediately replaced by the other register is necessary. These pseudoknots are the relatively stable examples mentioned previously, and we did not simulate full displacement in these cases but simply recorded them when they formed. One of the arms in the pseudoknot can also be displaced (in an inchworm fashion) by an alternative register.

Accurately measuring the transition rates between all registers is impractical owing to the large number of possibilities and the range of transition rates. Overall, however, initial alignments with >4 base pairs tend to undergo internal displacement to more strongly bound states, eventually reaching the full duplex, as shown in Supplementary Table S16. Misaligned duplexes of four base pairs also often detach instead. Internal displacement by a register with fewer base pairs than the original register is suppressed by the free-energy cost of breaking base pairs, although it is occasionally observed. We emphasize that the results in Supplementary Table S16 include examples of both inchworm and pseudoknot displacement: for instance, trajectories in which register −10 is displaced by register 2 typically involve a pseudoknot mechanism, passing through the short-lived intermediate −10,2, whereas displacement of register 2 by register 0 proceeds via the inchworm pathway.

As outlined in Supplementary Section S2D, we estimate that the most stable intermediates have a lifetime that is equal to the initial binding rate for concentrations on the order of a few micromolars. At significantly lower concentrations (which are often used in experiment), the actual time spent in these intermediates would then be negligible. Metastable states then simply provide alternative pathways for the second-order process of association, as shown schematically in Supplementary Figure S3. Alternative pathways increase the rate constant for binding of repetitive sequences (by a factor of five in our case: see Supplementary Section S4C). At higher concentrations, the finite lifetimes of intermediate states would be expected to be relevant. Repetitive sequences were also studied at 340.9 K see Supplementary Tables S17 and S18). At these temperatures, short duplexes melt quickly, and hence the probability that metastable structures are able to rearrange is reduced. Consequently, the rate of formation of the fully formed duplex falls off slightly faster with temperature than for the non-repetitive sequence: by a factor 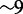 over the temperature range 300–340.9 K rather than 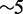. Internal displacement therefore provides another possible contribution to negative activation enthalpies in DNA duplex formation.

Having studied homopolymer RNA sequences experimentally, Pörschke (49) concluded that reaction kinetics was second-order, and therefore that mis-aligned sequences must be able to rearrange into the fully formed structure without fully detaching. He also proposed a mechanism essentially identical to inchworm displacement, ‘chain sliding by bulge diffusion’, by which this rearrangement might occur. Our results support this hypothesis and also extend it by identifying another mechanism for internal rearrangement of the duplexes. Unifying the processes under the concept of ‘internal displacement’ makes clear that both involve competition between two canonical duplex regions, emphasizes the analogy with conventional three-stranded displacement and differentiates them from alternative proposals such as ‘molecular slithering’ (26,27).

### Sequence-dependence of binding rates

Our simulations highlight two key phenomena involved in DNA oligomer hybridization: (i) initial contacts frequently dissociate before forming a full duplex and (ii) misaligned bonding can accelerate duplex formation through internal displacement. Both findings suggest possible mechanisms for sequence-dependence of DNA binding rates.

- In DNA, G-C base pairs are more stable than A-T. Hence, initial contacts between GC-rich sequences should be more stable and more likely to zip up following initial contact. If initial contacts form at approximately the same rate, duplexes with a greater density of G-C base pairs should form faster.
- The number, stability with respect to dissociation and ease of internal displacement of misaligned metastable states will vary greatly from sequence to sequence. Increasing any of these factors should result in faster duplex formation.

Systematic experimental investigations of the sequence dependence of DNA binding rates are not available. Zhang and Winfree (9), however, have probed sequence-dependent binding rates indirectly through toehold-mediated strand displacement. In the limit of long toeholds, the authors identified the displacement rate with the binding rate of toehold sequences. Their data showed a significant difference between the binding rates of GC-rich and AT-rich toeholds, with GC-rich toeholds causing faster displacement. To explore whether these differences can be attributed to the factors outlined here, we have simulated the association of eight-base duplexes using the sequences from (9), as given in Table 1. These simulations were performed using the Brownian thermostat with the sequence-dependent version of the model (more details are provided in Supplementary Sections S2B and S3F). The association rates are presented in Table 1. Also shown are the rates of displacement fitted by Zhang and Winfree (9) for G-C-rich, A-T-rich and average-strength toeholds, in the limit of long toehold lengths (we take our eight-base sequences from this source). These rates are assumed to reflect the association rates of the toeholds themselves (9). In agreement with (9), the GC-rich sequence forms duplexes fastest, followed by the average-strength sequence and finally the AT-rich sequence. The GC-rich sequence is faster than the AT-rich analogue by a factor of 7.4 in our simulations: Zhang and Winfree found a factor of 15 in the long-toehold limit. Interestingly, part of the factor of 7.4 in our model can be attributed to correctly aligned G-C base pairs being more stable than A-T equivalents, and part to an increased probability of binding via internal displacement. This conclusion follows from simulations in which internal displacement was suppressed by only allowing native base pairs: in this case, the GC-rich sequence was only 3.2 times as fast as the AT-rich variant.

**Table 1.** Binding rates of eight-base strands of different sequences at 298.15 K, relative to the G-C-rich case

Sequence	Type	Relative binding rate	Experimental rate constant /M^−1^s^−1^
Misaligned bonds allowed			
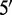 -CCCGCCGC- 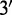	G-C-rich	1	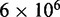
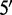 -TCTCCATG- 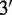	average-strength	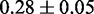	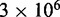
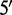 -ATTTATTA- 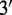	A-T-rich	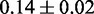	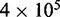
Misaligned bonds not allowed			
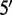 -CCCGCCGC- 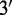	G-C-rich	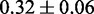	
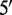 -ATTTATTA- 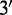	A-T-rich	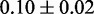	

Reaction rates were measured for the strands shown and their complements. Bimolecular rate constants are taken from the long-toehold limit of the fits in (9).

## CONCLUSIONS

We have studied DNA hybridization using a coarse-grained model, oxDNA, which was carefully optimized to represent both single and double-stranded DNA. Stiff, helical duplexes form in a realistic fashion from flexible single strands. By capturing these generic features of DNA, we predict a complex ensemble of transition pathways for association, without a single transition state with well-defined properties, and qualitatively distinct dynamics for different sequences. The association of a duplex occurs through the formation of initial contacts involving a small number of bases, followed by zippering of the remainder, as suggested previously (10,11,16,46). We go beyond this classic picture to show that initial contacts often dissociate, despite non-negligible attractive interactions, because their configurations are not conducive to full duplex formation and strands can detach before they equilibrate within the space of configurations defined by the secondary structure of the initial contacts. Thus, hybridization can fail even for interaction enthalpies which, if accessed from the equilibrium duplex ensemble, would overwhelmingly lead to duplex reformation. Moreover, if these processes were analysed at the secondary structure level, they would exhibit non-Markovian dynamics.

Increasing the temperature destabilizes initial contacts and lowers the drive to form more base pairs. The overall rate of association therefore decreases with temperature, resulting in a negative activation enthalpy if the results are interpreted through an Arrhenius model. At variance with the Arrhenius model, however, the effective activation enthalpy becomes more negative with increasing temperature, consistent with the fact that the strength of the initial contacts that are necessary to ensure duplex formation increases with temperature. Thus, the system does not possess a single, well-defined ‘transition state’ but a complex ensemble of transition pathways. This ensemble of pathways is further complicated by non-native interactions, which mean that systems can first form misaligned duplexes, and subsequently undergo internal displacement (rearranging without detaching) via *inchworm* or *pseudoknot* mechanisms to reach the fully formed duplex. At low reactant concentrations, these alternative pathways accelerate association. Further, owing to the principle of detailed balance, dissociation must also occur via internal displacement pathways, as well as via direct melting). As shown by the study of eight-base duplexes, sequences need not be perfectly repetitive for said pathways to be relevant. We note that for longer strands, the probability of binding in a misaligned fashion is higher. We would therefore expect these mechanisms to contribute strongly to the association of longer strands. Our observations of internal displacement mechanisms both support and extend the findings of Pörschke (49), who initially proposed a ‘defect diffusion’ model of internal rearrangement to explain kinetic data.

If initial contacts frequently fail, stronger contacts should prove more likely to succeed and thereby accelerate reaction rates. The rate of duplex formation through internal displacement will also depend strongly on the sequence. We tested the impact of these two proposed causes of sequence-dependent reaction rates and demonstrated their effect for short duplexes, finding agreement with the experimental data of Zhang and Winfree (9).

We therefore predict two main ways to modulate association rates. (i) More GC content will increase association rates of short sequences and (ii) for sequences of similar binding strength more repetitive sequences will bind faster because they allow additional internal displacement pathways. Our oxDNA simulations suggest that rates could differ by as much as an order of magnitude for oligomers of similar length. We note, however, that to resolve these effects clearly in experiment, care must be taken to avoid single-stranded secondary structure such as hairpin formation, which would open another (complex) avenue to modulating hybridization rates by changing sequence.

It is worth contrasting our results with those found for 3SPN.1, an important model that has also been used to study hybridization (25,26,50–52). These authors also observe complex kinetics but with significant differences. In particular, for non-repetitive sequences, the authors claim that duplexes typically form non-native contacts, before ‘snapping’ into the duplex state (26). Other studies with the same model have found that the strands ‘wind’ to form a double helix, then ‘slide’ past each other to reach appropriate alignment (50,52). Repetitive sequences form misaligned structures, which relax into the fully formed duplex by ‘slithering’ past each other (26,27). By contrast, the basic mechanism of duplex formation in oxDNA follows a clear nucleation and zippering pathway. Zippering occurs as bases from the relatively disordered single strands successively stack onto the growing duplex. Slithering, like internal displacement, allows misaligned duplexes to relax to the fully base-paired structure (26). The mechanisms, however, are distinct: internal displacement involves the formation of two separate, base-paired duplex regions that compete for bases, whereas slithering involves the sliding of strands past each other in a process ‘devoid of significant energy barriers’ (53). Both inchworm and pseudoknot mechanisms rely on single-stranded flexibility and hence will be suppressed in 3SPN.1. In oxDNA, slithering is suppressed, as it requires the system to pass through a double-helical state with no base pairing. Further discussion of the different mechanisms seen for 3SPN.1 and oxDNA is given in Supplementary Section S5.

OxDNA is a simplified model, and it is therefore appropriate to evaluate the robustness of our conclusions. First, the zipper-mechanism by which duplexes form relies on the existence of attractive interactions between bases, and the fact that the transition involves flexible single strands forming a stiff helical duplex. These are generic features of DNA that are well reproduced by oxDNA, and hence the conclusion is likely to be reliable. Second, the internal displacement mechanisms identified involve kinked and pseudoknotted intermediates that are well-established motifs in nucleic acid secondary structure (5,53). Moreover, oxDNA describes the kinetics of conventional strand displacement involving three strands well (N. Srinivas, T. E. Ouldridge, P. Šulc, J. Schaeffer, B. Yurke, A. A. Louis, J. P. K. Doye, and E. Winfree, submitted for publication). Finally, fast modes of relaxation for misaligned duplexes are consistent with the data of Pörschke (49). It is therefore likely that these pathways are genuine. Third, the frequent failure of initial contacts to form duplexes, despite the expected thermodynamic stability of extra base pairs, also relies on well-established differences between single strands and duplexes.

Nevertheless, it should be kept in mind that other contributions to the overall activation enthalpy may need to be taken into account. For example, we have not attempted to model the decrease of the viscosity of water with temperature, which may accelerate duplex formation at higher temperatures. It is also possible that interactions neglected or simplified in oxDNA play a role. For example, we do not consider microscopic barriers such as the disruption of solvating water molecules before hydrogen-bond formation. In Supplementary Section S4B, we show that initial contact between strands involves states with less intrastrand stacking on average than in the unbound ensemble. Some disrupted stacking is necessary for the strands to be in contact without being fully bound. This tendency contributes positively to the activation enthalpy—in our model, this effect is fairly small, but it would be larger if the structure of stacked single strands was less conducive to duplex formation. It is also conceivable that there are additional interactions between nucleotides (that are not evident in the duplex state) that tend to be disrupted before duplex formation, thereby contributing positively to the activation enthalpy. If said interactions do cause a positive activation enthalpy, however, the conclusion that CG-rich duplexes will form faster due to the stability of initial contacts should be robust, unless these interactions are systematically more obstructive for CG-rich sequences.

Experimental studies do not currently provide a consistent picture of hybridization kinetics. In particular, it is not clear whether the rate of duplex formation typically increases or decreases with temperature (corresponding to positive or negative activation enthalpies, respectively). We have, however, provided a physically reasonable justification for a negative contribution to activation enthalpies; that initial contacts are surprisingly likely to detach because the overall configuration of the two strands is not conducive to full duplex formation. This argument explains why, in Markov models constructed at the base-pair level, authors have postulated a ‘barrier’ to full duplex formation after the first few base pairs have formed (14), or made short sections of duplex detach quickly (10,11). Our simulations also suggest that if the failure of initial contacts is the cause of a negative activation enthalpy, precise experiments should show that this enthalpy becomes more negative with increasing temperature. Internal displacement can also enhance negative activation enthalpies.

By providing insight into the complex mechanisms by which two DNA strands associate, we can thus suggest ways to modulate strand association rate and thus choreograph the assembly and operation of DNA nanotechnology. Our findings arise from generic properties of DNA and the hybridization process, and we therefore expect them to apply to similar nucleic acid-based systems. For example, the folding of short RNA hairpins will likely involve much of the same biophysics, and processes such as internal displacement will be relevant. Future work will consider the role of single-stranded hairpins in determining reaction kinetics, and the consequences of internal displacement for the association of longer strands.

## SUPPLEMENTARY DATA

Supplementary Data are available at NAR Online.

## FUNDING

Engineering and Physical Sciences Research Council [EP/I001352/1]; and University College, Oxford. Funding for open access charge: EPSRC grant.

*Conflict of interest statement*. None declared.

## Supplementary Material

Supplementary Data
