# Supplementary material for: DNA hybridization kinetics: zippering, internal displacement and sequence dependence
Source: Nucleic Acids Res. 2013 Aug 8;41(19):8886–95. doi: 10.1093/nar/gkt687 (PMC3799446; doi:10.1093/nar/gkt687)
Supplement: Supplementary Data [file supp_41_19_8886__index.html]

DNA hybridization kinetics: zippering, internal displacement and sequence dependence — Supplementary Data 

# DNA hybridization kinetics: zippering, internal displacement and sequence dependence

## Supplementary Data

files

**Files in this Data Supplement:**

- Supplementary Data - pdf file
