# Supplementary material for: DNA hybridization kinetics: zippering, internal displacement and sequence dependence
Source: Nucleic Acids Res. 2013 Aug 8;41(19):8886–95. doi: 10.1093/nar/gkt687 (PMC3799446; doi:10.1093/nar/gkt687)
Supplement: Supplementary Data [file supp_gkt687_Binding_kinetics_NAR_sup.pdf]

Supplementary material for “DNA hybridisation kinetics: zippering, internal displacement and sequence dependence”

Thomas E. Ouldridge,[1] Petr Šulc,[1] Flavio Romano,[2] Jonathan P. K. Doye[2] and Ard A. Louis[1]
[1]*Rudolf Peierls Centre for Theoretical Physics, Department of Physics, University of Oxford, 1 Keble Road, Oxford, UK, OX1 3NP*
[2] *Physical & Theoretical Chemistry Laboratory, Department of Chemistry, University of Oxford, South Parks Road, Oxford, UK, OX1 3QZ*

CONTENTS

S1. The DNA model 1

S2. Simulation Methods 2
A. Langevin Dynamics 2
A.i. Forward flux sampling 3
B. Brownian Thermostat 4
C. VMMC 4
C.i. Umbrella sampling 4
D. Considerations of metastable states, fluxes and system size 4

S3. Simulation protocols 5
A. Hybridisation of non-repetitive duplexes 8
B. Hybridisation of repetitive duplexes 8
C. Internal displacement of misaligned repetitive sequences 8
D. Dissociation of misaligned repetitive sequences 8
E. Characterisation of the equilibrium ensemble 13
F. Sequence-dependence of association rate 13

S4. Results 13
A. Hybridisation of non-repetitive sequences 13
B. Characterisation of the ensemble of transition pathways, the equilibrium duplex ensemble and the equilibrium single-stranded ensemble 14
C. Hybridisation of repetitive sequences 16

S5. Detailed comparison of oxDNA with 3SPN.1 16

S1. THE DNA MODEL

OxDNA and its interaction potentials have been described in detail elsewhere.[1–3] The model represents DNA as a string of nucleotides, where each nucleotide (sugar, phosphate and base group) is a rigid body with interaction sites for backbone, stacking and hydrogen-bonding interactions. The potential energy of the system can be decomposed as

$$V = \sum_{\langle ij \rangle} \left(V_{\rm b.b.} + V_{\rm stack} + V'_{\rm exc} \right) + \sum_{i,j \notin \langle ij \rangle} \left(V_{\rm HB} + V_{\rm cr.st.} + V_{\rm exc} + V_{\rm cx.st.} \right), \quad (1)$$

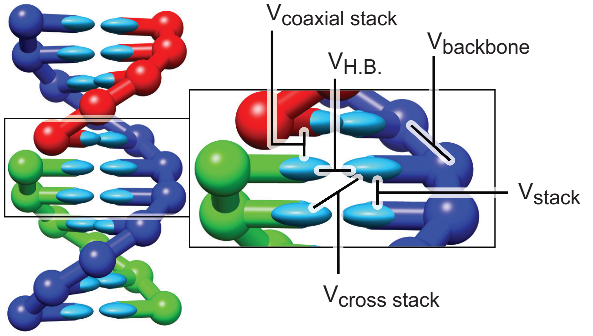

FIG. S1. A model DNA duplex, with stabilising interactions depicted schematically. The backbone sites are shown as spheres, the bases as ellipsoids. Backbone colouring indicates strand identity. All nucleotides also interact with repulsive excluded volume interactions. The coaxial stacking interaction acts like a stacking interaction between bases that are not immediate neighbours along the backbone of a strand. Taken from Ref. [4].

where the first sum is taken over all nucleotides that are nearest neighbours on the same strand and the second sum comprises all remaining pairs. The interactions between nucleotides are schematically shown in Fig. S1. The backbone potential $V_{\rm b.b.}$ is an isotropic spring that imposes a finite maximum distance between backbone sites of neighbours, mimicking the covalent bonds along the strand. The hydrogen bonding ($V_{\rm HB}$), cross stacking ($V_{\rm cr.st.}$), coaxial stacking ($V_{\rm cx.st.}$) and stacking interactions ($V_{\rm stack}$) are anisotropic and explicitly depend on the relative orientations of the nucleotides as well as the distance between the relevant interaction sites. This orientational dependence captures the planarity of bases, and helps drive the formation of helical duplexes. The coaxial stacking term is designed to capture stacking interactions between bases that are not immediate neighbours along the backbone of a strand. Bases and backbones also have excluded volume interactions $V_{\rm exc}$ or $V'_{\rm exc}$.

Hydrogen-bonding interactions are only possible between complementary (A-T and C-G) base pairs. In the sequence-dependent parameterisation, the strengths of interactions $V_{\rm stack}$ and $V_{\rm HB}$ further depend on the identity of the bases involved.[3] In the average model, $V_{\rm stack}$ is sequence-independent and $V_{\rm HB}$ is equivalent for (A-T and C-G) base pairs. Interactions were fitted to reproduce

melting temperatures and transition widths of oligonucleotides, as predicted by SantaLucia's nearest-neighbour model.[5] Note that our three dimensional model is significantly more complex than the nearest-neighbour model. We simply treat the latter as a high-quality fit to experimental data. Structural and mechanical properties of both double- and single-stranded DNA are also carefully taken into account in the fitting procedure. In DNA the double helical structure emerges because there is a length-scale mismatch between the preferred inter-base distance along the backbone, and the optimal separation of bases when stacking. It is exactly this feature that drives the helicity of oxDNA, rather than an imposed natural twist on the backbone. Overall, the emphasis in our derivation of oxDNA was on physics relevant for single-strand to duplex transitions. As discussed in the main text, oxDNA has been extensively tested for other DNA properties and systems to which it was not fitted. Our success in describing all these phenomena gives us confidence to use it to study the dynamics of hybridisation.

OxDNA was fitted to reproduce DNA behavior at salt concentration $[\mathrm{Na}^{+}] = 0.5\mathrm{M}$, where the electrostatic properties are strongly screened, and it is reasonable to incorporate them into a short-ranged excluded volume. The model therefore contains no further explicit electrostatic interactions. It should be noted that OxDNA neglects several features of DNA structure and interactions due to the high level of coarse-graining. Specifically, the double helix in the model is symmetrical rather than the grooves between the backbone sites having different sizes (i.e., major and minor grooving), and all four nucleotides have the same structure. These differences with real DNA mean that oxDNA will not be able to treat phenomena that depend sensitively, for example, on anisotropic elasticity, explicit salt ion effects, or the existence of major and minor grooving. However, these specific properties of DNA are unlikely to be critical to the general arguments we are making about hybridisation in this paper. Rather, it is the correct treatment of the basic mechanical properties of both single and double strands, together with the basic physics of hydrogen bonding and stacking that determines the emergent physical phenomena we are trying to describe.

We assume that the partial pressure of DNA in dilute solution is negligible relative to that of our implicit solvent. In this limit, it is appropriate to consider the coarse-grained DNA strands in the canonical ensemble, even when comparing to experiments performed at constant pressure.[6] We therefore use the terms enthalpy and energy interchangeably, with enthalpy most natural for comparisons with the experimental literature, and energy most natural when discussing the implementation of the model.

S2. SIMULATION METHODS

The thermodynamic properties of model DNA are given by averaging over the Boltzmann distribution

$$\rho(\mathbf{r}^N, \mathbf{p}^N, \mathbf{q}^N, \mathbf{L}^N) \;\propto\; \exp(-\beta\mathcal{H}(\mathbf{r}^N, \mathbf{p}^N, \mathbf{q}^N, \mathbf{L}^N)). \tag{2}$$

In this equation $\mathcal{H}$ is the system Hamiltonian, $\mathbf{r}$ and $\mathbf{q}$ are positional and orientational coordinates and $\mathbf{p}$ and $\mathbf{L}$ are linear and angular momenta. As terms in $\mathcal{H}$ containing $\mathbf{p}^N$ and $\mathbf{L}^N$ are separable and can be analytically integrated, the relative probability of a configuration is given by the Boltzmann factor of its potential energy, $\exp(-\beta V(\mathbf{r}^N, \mathbf{q}^N))$.

Inferring model kinetics necessitates an additional choice of dynamical algorithm. In this work we use Langevin Dynamics (LD) and a Brownian thermostat to measure dynamical properties. Virtual Move Monte Carlo (VMMC) is also used to calculate thermodynamic averages. For the dynamical algorithms, it is necessary to define a nucleotide mass which is taken as $m = 315.75\,\mathrm{Da}$ for all nucleotides.[2] For dynamical purposes, we treat the nucleotides as spherical with a moment of inertia $31.586\,\mathrm{Da\,nm}^2$.[2] The specification of mass, length and energy scales in the model together imply a time scale. For completeness, we will quote results in the supplementary material in terms of this time scale, although as discussed in the main text, relative times are likely to be more meaningful.

A. Langevin Dynamics

LD is a formalism for including random and dissipative forces due to an implicit solvent in a self-consistent manner so that solute particles move diffusively and the system samples from the Boltzmann distribution. Newton's equations of motion for the solute particles can be augmented with these forces and integrated to give dynamical trajectories. The results reported in this work were obtained using the quaternion-based algorithm of Davidchack *et al.*[7] To use LD, it is necessary to specify a friction tensor relating the drag forces experienced by a particle to its generalised momenta. We treat each nucleotide's interaction with the solvent as spherically symmetric, simplifying the friction tensors and leaving only two independent quantities, the linear and rotational damping coefficients γ and Γ. We choose values of $\gamma = 0.59\,\mathrm{ps}^{-1}$ and $\Gamma = 1.76\,\mathrm{ps}^{-1}$. These values produce overall diffusion coefficients of $D_{\mathrm{sim}} = 1.91 \times 10^{-9}\,\mathrm{m}^2\mathrm{s}^{-1}$ for a 14 base-pair duplex, higher than experimental measurements of $D_{\mathrm{exp}} = 1.19 \times 10^{-10}\,\mathrm{m}^2\mathrm{s}^{-1}$.[8] As discussed in the main text, accelerated diffusion is an advantageous aspect of coarse-grained modelling, allowing the simulations to access more complex processes. We show in Table S3 that using higher friction constants for the simple case of a non-repetitive sequence at 300 K slows down hybridisation, but does not qualitatively affect our results

otherwise: in particular, the tendency for initial contacts not to proceed to full duplex formation is preserved. LD Simulations in this work use a time step of 8.55 fs. This time step has been previously shown to reproduce the energies and kinetics of shorter time steps for the DNA model.[2]

A.i. Forward flux sampling

'Brute force' Langevin simulations are not always efficient enough to sample rare transitions. Forward flux sampling (FFS) allows the calculation of the flux between two local minima of free energy, and also samples from the trajectories that link the two minima (*reactive trajectories*).[9,10] Here we present a brief discussion of the FFS method in general. Our particular implementation will be discussed later.

The term 'flux' from (meta)stable state A to state B has the following definition.

> Given an infinitely long simulation in which many transitions are observed, the flux of trajectories from A to B is $\Phi_{AB} = N_{AB}/(\tau f_A)$, where N_{AB} is the number of times the simulation leaves A and then reaches B, τ is the total time simulated and f_A is the fraction of the total time simulated for which state A has been more recently visited than state B.

The concept of flux is therefore a generalisation of a transition rate for processes that are not instantaneous: it incorporates the time spent in intermediate states between A and B. Subtleties relating to the inference of rates from our simulations are discussed in Appendix S2 D.

To use FFS, we require an order parameter Q which measures the extent of the reaction, such that non-intersecting interfaces, λ^n_{n-1} can be drawn between consecutive values of Q. Initially, simulations are performed that begin in the lowest value of Q (which we define as $Q = -2$), and the flux of trajectories crossing the surface λ^0_{-1} (for the first time since leaving $Q = -2$) is measured. We define the lowest value of Q as $Q = -2$ because the simulation procedure is distinct for $Q > 0$.

The total flux of trajectories from $Q = -2$ to the alternative minima ($Q = Q_{\max}$) is then calculated as the flux across λ^0_{-1} from $Q = -2$, multiplied by the conditional probability that these trajectories reach $Q = Q_{\max}$ before returning to to $Q = -2$. This probability can be factorised into the product of the probabilities of trajectories starting from the interface λ^Q_{Q-1} reaching the interface λ^{Q+1}_Q before returning to $Q = -2$ to yield:

$$P(\lambda^{Q_{\max}}_{Q_{\max}-1}|\lambda^0_{-1}) = \prod_{Q=1}^{Q_{\max}} P(\lambda^Q_{Q-1}|\lambda^{Q-1}_{Q-2}). \tag{3}$$

In this work we use two distinct approaches to evaluating the product in equation 3, known as *direct* FFS and *Rosenbluth* FFS. Direct FFS proceeds by randomly loading microstates at the interface λ^0_{-1} saved during the calculation of the initial flux, and using these as starting configurations from which to estimate $P(\lambda^1_0|\lambda^0_{-1})$ by direct simulation. The process is then iterated for successive interfaces, using the successful trajectories from the previous interface as initial configurations for the next, allowing the estimation of $P(\lambda^Q_{Q-1}|\lambda^{Q-1}_{Q-2})$ for all relevant values of Q. Thus the flux from from $Q = -2$ to $Q = Q_{\max}$ can be calculated, and the trajectories obtained sample from the distribution of reactive trajectories. Rosenbluth FFS is an alternative approach in which, instead of successively performing a large number of simulations at every interface, individual reactive trajectories are generated independently by performing a small number of simulations at each interface for each trajectory. If multiple attempts are successful at a given interface, one is chosen at random and the rest are discarded. The contrasting approaches are illustrated in Fig. S2. Note that extracting the flux and ensemble of reactive trajectories requires a re-weighting procedure for Rosenbluth FFS, due to the fact that some trajectories are discarded.[9,10]

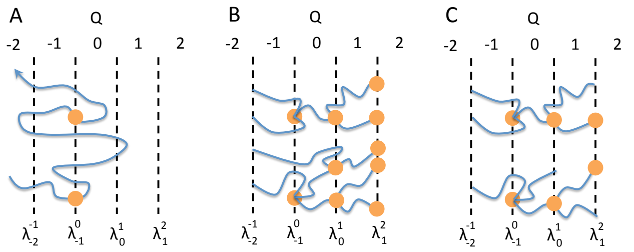

FIG. S2. Schematic illustrations of FFS. An order parameter Q is defined with interfaces λ separating distinct value of Q. We are interested in measuring the flux from $Q = -2$ to $Q = 2$ in this example. A) The initial measurement of flux across the interface λ^0_{-1}. Orange dots indicate the crossings that contribute to the flux, and also the states used to launch subsequent stages of simulation. B) Direct FFS involves randomly launching many trajectories from the interface λ^0_{-1}, and measuring the probability of reaching λ^1_0 before returning to λ^{-1}_{-2}. This procedure is then repeated for successive interfaces, resulting in branched trajectories. C) Rosenbluth FFS grows complete reactive trajectories in isolation: for each point on the previous interface, a fixed number of trajectories are launched. If one or more successfully reach the next interface, a single case is chosen at random and the rest are discarded.

Direct FFS naturally produces branched trajectories, whereas Rosenbluth FFS does not. Rosenbluth sampling thus provides an equally good sampling of the initial stages of a reaction as the final stages, unlike direct FFS which samples the later stages in more detail. Unfortunately, however, Rosenbluth sampling is less efficient in obtaining reactive trajectories due to a tendency to generate successful simulations that are then discarded (to avoid branching). For these reasons we used Rosenbluth FFS where possible, but used direct FFS for the most difficult simulations.

The random error associated with FFS simulations can

be estimated in the following way. Measurements of the flux across λ^0_{-1} are performed using N independent simulations. The daughter trajectories of any one of these initial simulations therefore give an independent estimate of the flux. We report the error as $\sigma/\sqrt{N-1}$, where σ^2 is the variance of the N independent estimates.

It is sensible to check that the FFS measurements are reasonable. This is possible because, during the sampling of the flux across interface λ^0_{-1}, successful transitions to $Q_{\rm max}$ are occasionally observed for the simplest cases. For example, during the simulation of the 14-base strands in which only native contacts were permitted, six transitions were observed in 72 μs, giving a rate of $\sim 8\times10^4\,{\rm s}^{-1}$, consistent with the estimate of $(6.23\pm0.47)\times10^4\,{\rm s}^{-1}$ from FFS (Table S13).

B. Brownian Thermostat

A simple alternative to LD is to use an algorithm which evolves the system according to Newton's equations for a fixed length of time, then resample a fraction of the velocities and angular velocities from the Maxwell distribution. We refer to this type of algorithm as a Brownain thermostat, and our simulations were performed using the thermostat described in Ref.[11]. The simulation algorithm performs Verlet integration[12] for a given number of steps $N_{\rm Newt}$, then resets the velocity of each nucleotide with probability p_v and the angular velocity of each nucleotide with a probability p_ω. The newly assigned velocities and angular velocities are drawn from the Boltzmann distribution. In our simulations, we chose $p_v = 0.02$, $p_\omega = 0.0068$ and $N_{\rm Newt} = 103$. On time scales longer than $N_{\rm Newt}\delta t/p_v$, where δt is the integration time step, the dynamics is diffusive. Using $\delta t = 8.53$ fs, 14-base strands of DNA have a diffusion coefficient of $7.6 \times 10^{-8}\,{\rm m^2s^{-1}}$ using this algorithm, higher than the $D_{\rm sim} = 1.91 \times 10^{-9}\,{\rm m^2s^{-1}}$ measured for our LD algorithm.

C. VMMC

VMMC[13,14] is a Monte Carlo technique effective for diluted systems with strong, directional interactions such as our DNA model. The algorithm generates a series of configurations of a system that are drawn from the Boltzmann distribution. The algorithm moves from one configuration to the next by attempting moves of clusters that are generated in a manner that reflects local potential energy gradients in the system. Trial moves are accepted with a probability that ensures the system samples from the canonical ensemble. By moving clusters of strongly interacting particles, the algorithm is able to equilibrate model DNA systems much faster than simpler Monte Carlo algorithms. To use VMMC, it is necessary to select 'seed' moves of a single particle: the resultant energy changes are used to generate the cluster. For all VMMC simulations reported here, the seed moves were:

- Rotation of a nucleotide about its backbone site, with the axis chosen from a uniform random distribution and the angle from a normal distribution with mean of zero and a standard deviation of 0.12 radians.

- Translation of a nucleotide with the direction chosen from a uniform random distribution and the distance from a normal distribution with mean of zero and a standard deviation of 1.02 Å.

C.i. Umbrella sampling

Despite the simplicity of the model and the efficiency of VMMC, many processes are still slow to equilibrate due to the presence of large free-energy barriers. These barriers can be artificially flattened, and equilibration enhanced, by incorporating an additional biasing weight $W(\mathbf{r}^N, \mathbf{q}^N)$[15]. In this approach, known as umbrella sampling, $W(\mathbf{r}^N, \mathbf{q}^N)$ is chosen to favour the states of high free energy, and the expectation of any variable A can be extracted as

$$\langle A \rangle = \frac{\langle A(\mathbf{r}^N, \mathbf{q}^N)/W(\mathbf{r}^N, \mathbf{q}^N)\rangle_W}{\langle 1/W(\mathbf{r}^N, \mathbf{q}^N)\rangle_W}. \qquad (4)$$

Here $\langle\rangle_W$ indicates sampling from the ensemble in which states have a relative probability $W(\mathbf{r}^N, \mathbf{q}^N)\exp(-\beta V(\mathbf{r}^N, \mathbf{q}^N))$.

D. Considerations of metastable states, fluxes and system size

FFS is not effective at simulating transitions with long-lived metastable intermediates, as the process of escaping these intermediates must be directly simulated through brute-force methods. Such long lived intermediates are present with the repetitive sequences we have studied. FFS can, however, be used to simulate separately the flux of trajectories into and out of these states. This data can then be used to estimate overall reaction kinetics, by constructing a model such as that illustrated in Fig. S3 A. Performing this calculation implicitly assumes that the system equilibrates within the metastable intermediate states before making another transition.

In our work, we have employed this approximation to study repetitive sequences, defining a number of misbonded intermediates and calculating the fluxes between them. The states considered are generally long-lived, show limited heterogeneity in structure within a state and are separated by significant free-energy barriers from other states. Thus the quasi-equilibrium assumption is reasonable.

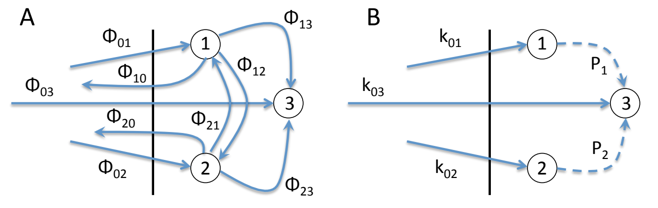

FIG. S3. Reaction kinetics in the presence of metastable intermediates. A) Fluxes Φ contributing to the kinetics of formation of bound state "3" from the unbound ensemble "0" (taken as the left-hand-side of the diagram), in the presence of two metastable intermediates "1" and "2". B) Simplified picture used to interpret our results, in which each bound state is formed with a certain rate, and states 1 and 2 convert to the fully bound state 3 instantaneously with probabilities P_1 and P_2. The overall reaction rate is then $k_{03} + k_{01}P_1 + k_{02}P_2$.

Formation of either a metastable intermediate or the fully bonded state occurs in a short time scale after initial contact has been made, relative to the overall time spent in the single-stranded ensemble. Thus it is sensible to interpret the measured fluxes from the unbound ensemble directly as instantaneous reaction rates. Due to the existence of metastable intermediates for repetitive sequences, however, the *overall* process of moving from unbound to fully bound ensembles is not effectively instantaneous on the time scales that strands encounter one another through diffusion. In our simulations, a significant amount of time can be spent in misaligned registers or pseudoknots. In principle, this makes the definition of an overall reaction rate problematic.

For computational tractability, however, we have simulated systems of two DNA strands with a relatively small volume, giving strand concentrations of $\sim 100\,\mu$M. This is much higher than typical experiments – for instance, Zhang and Winfree[16] used concentrations of order 1 nM. In our system, all interactions are short ranged and hence the strands behave approximately ideally unless they come into close contact. Thus the effect of diluting the system is trivial: using a cell of twice the volume would halve the rate at which strands came into contact. Dilution should not, however, affect the rate at which strands dissociate or internally rearrange once attached, and nor will it affect the probability of choosing a given pathway out of a misbonded configuration. Therefore, although in our simulations a significant amount of time can be spent in metastable intermediates, at the much lower concentrations relevant to experiment these times will be negligible compared to the diffusional timescales required to make contact.

When we compare relative rates to experiment, the time required to form an initial contact should scale with the dilution, and thus *relative* rates of initial attachment measured in simulation are directly comparable to experiment. Any time spent in metastable intermediates, however, should be subtracted from the total time to reach a duplex from the single-stranded state in order to make a fair comparison. Practically, this means that when analysing the repetitive sequence, we simply use the measured fluxes out of each metastable state to calculate the probability that a given intermediate will progress to the fully formed duplex before dissociating. The overall reaction rate $k_{\rm on}$ is then

$$k_{\rm on} = \sum_i k_i P_i, \tag{5}$$

where the sum runs over all bound states i, k_i is the formation rate of i from the unbound ensemble and P_i is the probability that such a state will convert to the fully-formed structure before dissociating. We illustrate this analysis schematically in Fig. S3 B. For the sequence-dependent study, we simply subtract the time in which the two strands have one or more base pairs from the total time that it takes to reach the fully-bound state. We thus compensate for the fact that rearrangement is a significant contribution to reaction times at our concentrations, but not in the dilute limit.

At high enough concentrations of reactants, of course, this approximation would break down. From our data (Table S16)), we estimate that the metastable misbonded configurations with the longest lifetimes at 300 K are those in register ± 2, with a 'decay' rate of $\sim 2 \times 10^4\,{\rm s}^{-1}$, or the more stable pseudoknot structures, which (from the amount of base pairing that is possible in each arm) we would expect to resolve at a rate at least as fast as the melting of a 6-bp duplex ($\sim 10^4\,{\rm s}^{-1}$). Initial binding rates, as measured by our model (Table S15), are $\sim 4 \times 10^5\,{\rm s}^{-1}$ at $100\,\mu$M. Therefore, at concentrations below $\sim 1\,\mu$M, neglecting the time spent in intermediates is likely to be reasonable, but at significantly larger concentrations we would expect non-second order effects to be relevant. At higher temperature, the time spent in intermediates drops (see Table S18) and we would expect the approximation to be valid at higher concentrations.

S3. SIMULATION PROTOCOLS

In this section we discuss the implementation of the algorithms of Appendix S2 for the specific systems studied in this work, and present some raw data from the simulations which would allow reproduction of the results. Processed data is presented in Appendix S4. To facilitate the discussion, we introduce the following concepts.

- *Native* base pairs are those that are expected to form in the fully-bound structure.
- For repetitive sequences, a number of metastable intermediates exist. Some of these are simply misaligned structures, which can be unambiguously defined by their *register*: a register of r_1 corresponds to bases pairing with a partner offset by r_1 bases in the $5'$ direction from their native partner. For non-repetitive sequences, only $r_1 = 0$ is relevant. The maximum number of base pairs in a register r_1 is $14 - |r_1|$.

Order parameter Q	Separation d/nm	Nearly-formed base pairs n	Set of base pairs A with $E < E_A$	Set of base pairs B with $E < E_B$
$Q = -2$	$d > 5.11$	$\sim$	$\sim$	$\sim$
$Q = -1$	$5.11 \geq d > 3.42$	$\sim$	$\sim$	$\sim$
$Q = 0$	$3.42 \geq d > 2.56$	$\sim$	$\sim$	$\sim$
$Q = 1$	$2.56 \geq d > 1.71$	$\sim$	$\sim$	$\sim$
$Q = 2$	$1.71 \geq d > 0.85$	$\sim$	$\sim$	$\sim$
$Q = 3$	$d \leq 0.85$	$n = 0$	$\|A\| = 0$	$\|B\| = 0$
$Q = 4$	$\sim$	$n \geq 1$	$\|A\| = 0$	$\|B\| = 0$
$Q = 5$	$\sim$	$\sim$	$(\|A\| \geq 1$ & $\|B\| = 0)$ or $(\|A\| = 1$ & $\|B\| = 1)$	
$Q = 6$	$\sim$	$\|A\| \geq 2$ & $\|B\| \geq 1$ & $(A \notin S_r$ or $B \notin S_r$ or $n \neq 0^*)$		
$Q = 7$	$\sim$	$n = 0^*$	$A \in S_r$	$B \in S_r$

TABLE S1. Order parameter definitions for FFS simulations of binding. $|A|$ is the number of base pairs in set A. $A \in S_r$ indicates that the set of interactions A is in the set of sets S_r that defines a target state of the simulations. For non-repetitive systems, only S_0 is relevant. For repetitive sequences at $T = 300\,$K, misaligned structures with at least 4 base pairs $(14 - |r_1| \geq 4)$ are considered, and the pseudoknots $\{r_1, r_2\} = \{-4, 8\}$, $\{-6, 6\}$, $\{-6, 8\}$, $\{-8, 4\}$ and $\{-8, 6\}$. For repetitive sequences at $T = 340.9\,$K, only registers with at least eight base pairs are considered $(14 - |r_1| \geq 8)$, and pseudoknots are not. $n = 0^*$ indicates that nearly formed base pairs are forbidden, except in pseudoknot cases when nearly-formed base pairs from the relevant registers are allowed. The symbol '$\sim$' indicates that no restriction is placed on this generalised coordinate except those that follow implicitly from the other requirements. We use two energy cutoffs to monitor base-pairing: $E_A = -1.43\,\mathrm{kcal\,mol^{-1}}$ and $E_B = -1.79\,\mathrm{kcal\,mol^{-1}}$.

	Temperature/K				
	300	312.5	326.1	340.9	300′
Number of simulations for flux across λ^0_{-1}	20	20	20	20	20
Initialisation time per simulation /ns	860	860	860	860	860
Crossings of λ^0_{-1} (time taken/μs)	8106 (75)	8059 (69)	7948 (58)	8041 (50)	7854 (72)
Flux across λ^0_{-1} /μs^{-1}	108 ± 4	116 ± 2	138 ± 5	160 ± 8	109 ± 4
Total trajectories started from λ^0_{-1}	12000	4000	4000	4000	6000
Target interface	Success probability (attempts per trajectory)				
λ^1_0	0.427 ± 0.005 (5)	0.420 ± 0.009 (5)	0.418 ± 0.008 (5)	0.427 ± 0.008 (5)	0.431 ± 0.006 (5)
λ^2_1	0.496 ± 0.003 (5)	0.517 ± 0.006 (5)	0.495 ± 0.007 (5)	0.514 ± 0.008 (5)	0.504 ± 0.004 (5)
λ^3_2	0.521 ± 0.004 (5)	0.528 ± 0.008 (5)	0.524 ± 0.006 (5)	0.545 ± 0.006 (5)	0.514 ± 0.006 (5)
λ^4_3	0.534 ± 0.004 (5)	0.567 ± 0.008 (5)	0.580 ± 0.009 (5)	0.609 ± 0.008 (5)	0.275 ± 0.006 (5)
λ^5_4	0.146 ± 0.002 (20)	0.154 ± 0.004 (40)	0.165 ± 0.003 (40)	0.161 ± 0.004 (40)	0.0685 ± 0.003 (20)
λ^6_5	0.252 ± 0.01 (5)	0.182 ± 0.01 (10)	0.118 ± 0.008 (10)	0.0908 ± 0.009 (10)	0.418 ± 0.02 (5)
λ^7_6	0.326 ± 0.02 (5)	0.265 ± 0.03 (10)	0.181 ± 0.02 (10)	0.0778 ± 0.01 (10)	0.648 ± 0.02 (5)
Total reactive trajectories found	1535	608	485	244	829

TABLE S2. Details of Rosenbluth FFS simulations performed to estimate the kinetics of binding for non-repetitive sequences at a range of temperatures (300′ corresponds to simulations in which only native bonds had a non-zero interaction). The top half of the table reports the initial simulations of flux across the λ^0_{-1} interface. The bottom half contains the simulation data from the later stages: the total number of trajectories initiated at λ^0_{-1}, the success rates of attempts to reach the next interface and the number of attempts per trajectory performed at each interface.

Friction coefficients γ /ps^{-1}, Γ /ps^{-1}	0.59, 1.76	5.9, 17.6
Difffusion coefficient / m^{2}s^{-1}	1.91×10^{-9}	1.91×10^{-10}
Number of simulations for flux across λ_{-1}^0	20	20
Initialisation time per simulation /ns	860	860
Crossings of λ_{-1}^0 (time taken/μs)	8106 (75)	7734 (479)
Flux across λ_{-1}^0 /μs^{-1}	108 ± 4	16.2 ± 0.5
Total trajectories loaded from λ_{-1}^0	12000	6000
Target interface	Success probability (attempts per trajectory)	
λ_0^1	0.427 ± 0.005 (5)	0.394 ± 0.005 (5)
λ_1^2	0.496 ± 0.003 (5)	0.476 ± 0.006 (5)
λ_2^3	0.521 ± 0.004 (5)	0.513 ± 0.007 (5)
λ_3^4	0.534 ± 0.004 (5)	0.630 ± 0.005 (5)
λ_4^5	0.146 ± 0.002 (20)	0.258 ± 0.003 (20)
λ_5^6	0.252 ± 0.01 (5)	0.190 ± 0.008 (5)
λ_6^7	0.326 ± 0.02 (5)	0.267 ± 0.02 (5)
Total reactive trajectories found	1535	584

TABLE S3. Comparison of Rosenbluth FFS simulations of hybridisation for non-repetitive sequences at 300 K for implementations of LD with different friction constants. $\gamma = 0.59\,\mathrm{ps}^{-1}$ and $\Gamma = 1.76\,\mathrm{ps}^{-1}$ are the standard values used in this work. The top half of the table reports the initial simulations of flux across the λ_{-1}^0 interface. The bottom half contains the simulation data from the later stages: the total number of trajectories initiated at λ_{-1}^0, the success rates of attempts to reach the next interface and the number of attempts per trajectory performed at each interface.

	Temperature/K	
	300	340.9
Number of simulations for flux across λ_{-1}^0	50	50
Initialisation time per simulation /ns	860	860
Crossings of λ_{-1}^0 (time taken/μs)	19776 (186)	19973 (133)
Flux across λ_{-1}^0 /μs^{-1}	106 ± 2	150 ± 3
Total trajectories loaded from λ_{-1}^0	15000	6000
Target interface	Success probability (attempts per trajectory)	
λ_0^1	0.434 ± 0.004 (5)	0.417 ± 0.004 (5)
λ_1^2	0.505 ± 0.003 (5)	0.504 ± 0.005 (5)
λ_2^3	0.519 ± 0.003 (5)	0.532 ± 0.005 (5)
λ_3^4	0.633 ± 0.003 (5)	0.707 ± 0.006 (5)
λ_4^5	0.185 ± 0.002 (20)	0.210 ± 0.003 (40)
λ_5^6	0.442 ± 0.007 (5)	0.211 ± 0.009 (10)
λ_6^7	0.642 ± 0.009 (5)	0.143 ± 0.009 (10)
Total reactive trajectories found	5523	1207

TABLE S4. Details of Rosenbluth FFS simulations performed to estimate the kinetics of binding for repetitive sequences at 300 K and 340.9 K. The top half of the table reports the initial simulations of flux across the λ_{-1}^0 interface. The bottom half contains the simulation data from the later stages: the total number of trajectories initiated at λ_{-1}^0, the success rates of attempts to reach the next interface and the number of attempts per trajectory performed at each interface.

- Other structures involve two registers $\{r_1, r_2\}$ in a pseudoknotted configuration. We found that $\{r_1, r_2\} = \{-4, 8\}$, $\{-6, 6\}$, $\{-6, 8\}$, $\{-8, 4\}$ and $\{-8, 6\}$ were long-lived metastable states, as displacement of one arm by the other is limited.

- Given criteria for identifying base-pairing interactions, a given state of the system will have a set of interactions A. Let A be in the set of sets S_r if A is characteristic of the bonding pattern in register $r = r_1$ or pseudoknot $r = \{r_1, r_2\}$. For purely misaligned structures, we take A to be in S_{r_1} if every possible base pair in register r_1 is present, with no other interactions. Pseudoknots have a greater degree of heterogeneity (base pairs can be exchanged between registers). For our purposes, a set of interactions A is in S_{r_1,r_2} if and only if each of the registers has at least 6 interactions, and no other interactions are present.

- The separation $d_{\min}$ is the minimum distance between hydrogen-bonding sites over all pairs of bases in the two strands.

- One way of identifying interactions is through the *nearly formed* base pair. A potential base pair between the strands is counted as *nearly formed* when the conditions outlined below hold.
  - The separation of hydrogen-bonding sites is ≤ 0.85 nm.
  - The hydrogen-bonding potential consists of a separation dependent factor multiplied by a number of modulating angular factors. At most one of these factors that contributes mul-

Order parameter Q	Set of base pairs A with $E < E_A$	Nearly-formed base pairs n	Set of base pairs B with $E < E_B$	Set of base pairs C not in S_{r_0} with $E < E_A$	Set of base pairs D not in S_{r_0} with $E < E_B$
$Q = -2$	$A \in S_{r_0}$	$n = 0$	$B \in S_{r_0}$	$\sim$	$\sim$
$Q = -1$	$\sim$	$(B \in S_{r_0}$ & $n \neq 0)$ or $B \notin S_{r_0}$		$\|C\| = 0$	$\|D\| = 0$
$Q = 0$	$\sim$	$\sim$	$\sim$	$(\|C\| \geq 1$ & $\|D\| = 0)$ or $(\|C\| = 1$ & $\|D\| = 1)$	
$Q = 1$	$\sim$	$\sim$	$\sim$	$(\|C\| \geq 2$ & $\|D\| = 1)$ or $(\|C\| = 2$ & $\|D\| = 2)$	
$Q = 2$	$\sim$	$\sim$	$\sim$	$(\|C\| \geq 3$ & $\|D\| = 2)$ or $(\|C\| = 3$ & $\|D\| = 3)$	
$Q = 3$	$A \notin S_{r'}$ or $n \neq 0^*$ or $B \notin S_{r'}$			$\|C\| \geq 4$	$\|D\| \geq 3$
$Q = 4$	$A \in S_{r'}$	$n = 0^*$	$B \in S_{r'}$	$\sim$	$\sim$

TABLE S5. Order parameter definitions for FFS simulations of internal displacement. $|A|$ is the number of base pairs in set A. $A \in S_r$ indicates that the set of interactions A is in the set of sets S_r. S_{r_0} is the set corresponding to the initial (misaligned) state of the system. $S_{r'}$ is the set corresponding to any *other* possible target state. For repetitive sequences at $T = 300\,\mathrm{K}$, misaligned structures with at least four base pairs $(14 - |r_1| \geq 4)$ are considered, and the pseudoknots $\{r_1, r_2\} = \{-4, 8\}$, $\{-6, 6\}$, $\{-6, 8\}$, $\{-8, 4\}$ and $\{-8, 6\}$. For repetitive sequences at $T = 340.9\,\mathrm{K}$, only registers with at least eight base pairs $(14 - |r_1| \geq 8)$ are considered, and pseudoknots are not. $n = 0^*$ indicates that nearly formed base pairs are forbidden, except in pseudoknot cases when nearly-formed base pairs from the relevant registers are allowed. The symbol '$\sim$' indicates that no restriction is placed on this generalised coordinate except those that follow implicitly from the other requirements. We use two energy cutoffs to monitor base-pairing: $E_A = -1.43\,\mathrm{kcal\,mol^{-1}}$ and $E_B = -1.79\,\mathrm{kcal\,mol^{-1}}$. During simulations, the system was also monitored to check for dissociation (when $d_{\min} > 5.11\,\mathrm{nm}$).

tiplicatively to the hydrogen-bonding energy is zero.

– The hydrogen-bonding interaction is less negative (weaker) than $-1.43\,\mathrm{kcal\,mol^{-1}}$. Typical hydrogen bonds have enthalpies of $\sim -3.6\,\mathrm{kcal\,mol^{-1}}$.

Physically, these conditions mean that the bases are close and fairly well aligned, but not forming a strong base pair.

A. Hybridisation of non-repetitive duplexes

Studies of hybridisation were performed using Rosenbluth FFS. The order parameter Q, as detailed in Table S1, combines separation-based and interaction strength metrics. Its complicated form is designed to optimise sampling, and reduce the possibility of two interfaces being crossed in a single integration time step (this is aided by the use of two different energy cutoffs for quantifying the degree of base-pairing). The condition for $Q = 7$ indicates a bound state in this work, and will be relevant in other cases. For the repetitive sequences, we simultaneously measure the flux into a number of possible binding registers. Data from the simulations are given in Table S2.

B. Hybridisation of repetitive duplexes

Measurements of the initial stage of attachment were performed exactly analogously to the non-repetitive duplexes, and the order parameter is outlined in Table S1. In this case, the flux of trajectories into several metastable structures was measured simultaneously. At 300 K, purely misaligned structures with at least four base pairs ($r_1 = 0, \pm 2, \pm 4, \pm 6, \pm 8, \pm 10$) were considered, as well as the pseudoknots $\{r_1, r_2\} = \{-4, 8\}$, $\{-6, 6\}$, $\{-6, 8\}$, $\{-8, 4\}$ and $\{-8, 6\}$. At 340.9 K, only misaligned structures with at least eight base pairs were considered as metastable targets, because other structures melt rapidly and require no explicit treatment. Details of the simulations are given in Table S4.

C. Internal displacement of misaligned repetitive sequences

Measurements of internal displacement were performed using Rosenbluth FFS. The order parameter is outlined in Table S5. At 300 K, we consider the flux of trajectories from any misaligned structure to any other alignment with at least 4 base pairs, as well as the metastable pseudoknot states $\{r_1, r_2\} = \{-4, 8\}$, $\{-6, 6\}$, $\{-6, 8\}$, $\{-8, 4\}$ and $\{-8, 6\}$. The relaxation of these metastable pseudoknots proved to be too difficult to simulate reliably. At 340.9 K, only alignments with at least eight base pairs were considered. Simulations were monitored to check for strand dissociation: trajectories that resulted in dissociation were ended and counted as 'failures' for the purposes of measuring the flux of internal displacement. Further details of the simulations are given in Table S6.

D. Dissociation of misaligned repetitive sequences

Measurements of dissociation of misaligned structures were performed using direct FFS. The order parameter is outlined in Tables S8 and S9. At 300 K, dissociation was studied for registers $r_1 = \pm 8$ and ± 10. Longer mis-

**A**	Initial register				
	2	4	6	8	10
Number of simulations for flux across λ^0_{-1}	10	10	10	10	10
Initialisation time per simulation /ns	8.6	8.6	8.6	8.6	8.6
Crossings of λ^0_{-1} (time taken/μs)	1000 (39)	1000 (54)	1000 (52)	1000 (46)	1000 (41)
Flux across λ^0_{-1} /μs^{-1}	25.9 ± 0.6	18.7 ± 0.8	19.4 ± 0.7	21.5 ± 0.7	24.4 ± 1
Total trajectories started from λ^0_{-1}	10000	5000	5000	6000	5000
Target interface	Success probability (attempts per trajectory)				
λ^1_0	0.155 ± 0.006 (20)	0.258 ± 0.01 (20)	0.275 ± 0.01 (20)	0.260 ± 0.01 (10)	0.289 ± 0.01 (10)
λ^2_1	0.239 ± 0.02 (20)	0.232 ± 0.02 (20)	0.224 ± 0.01 (20)	0.261 ± 0.02 (10)	0.401 ± 0.02 (3)
λ^3_2	0.170 ± 0.02 (20)	0.235 ± 0.02 (20)	0.248 ± 0.01 (20)	0.504 ± 0.02 (10)	0.860 ± 0.02 (3)
λ^4_3	0.115 ± 0.008 (6)	0.193 ± 0.008 (6)	0.355 ± 0.03 (6)	0.807 ± 0.03 (10)	0.971 ± 0.005 (2)
Total reactive trajectories found	775	1052	1365	1309	1245

**B**	Initial register				
	−2	−4	−6	−8	−10
Number of simulations for flux across λ^0_{-1}	10	10	10	10	10
Initialisation time per simulation /ns	8.6	8.6	8.6	8.6	8.6
Crossings of λ^0_{-1} (time taken/μs)	1000 (41)	1000 (54)	1000 (54)	1000 (50)	1000 (42)
Flux across λ^0_{-1} /μs^{-1}	24.5 ± 0.7	18.4 ± 0.6	18.6 ± 0.6	20.2 ± 0.6	24.1 ± 0.5
Total trajectories started from λ^0_{-1}	10000	10000	10000	8500	8500
Target interface	Success probability (attempts per trajectory)				
λ^1_0	0.182 ± 0.009 (20)	0.266 ± 0.01 (20)	0.273 ± 0.02 (20)	0.276 ± 0.01 (10)	0.308 ± 0.009 (10)
λ^2_1	0.240 ± 0.02 (20)	0.216 ± 0.02 (20)	0.243 ± 0.02 (20)	0.282 ± 0.01 (10)	0.436 ± 0.02 (3)
λ^3_2	0.221 ± 0.03(20)	0.212 ± 0.01 (20)	0.264 ± 0.02 (20)	0.482 ± 0.03 (10)	0.863 ± 0.01 (3)
λ^4_3	0.112 ± 0.003 (6)	0.203 ± 0.007 (6)	0.344 ± 0.04 (6)	0.774 ± 0.02 (10)	0.960 ± 0.005 (2)
Total reactive trajectories found	823	2139	2731	1957	2281

TABLE S6. Details of Rosenbluth FFS simulations performed to estimate the kinetics of internal displacement at 300 K, for positive (A) and negative (B) registers. The top half of each table reports the initial simulations of flux across the λ^0_{-1} interface. The bottom half contains the simulation data from the later stages: the total number of trajectories initiated at λ^0_{-1}, the success rates of attempts to reach the next interface and the number of attempts per trajectory performed at each interface.

	Initial register		
	2	4	6
Number of simulations for flux across λ_{-1}^0	10	10	10
Initialisation time per simulation /ns	8.6	8.6	8.6
Crossings of λ_{-1}^0 (time taken /μs)	1000 (11)	1000 (11)	1000 (10)
Flux across λ_{-1}^0 /μs^{-1}	92 ± 2	93 ± 3	101 ± 3
Total trajectories started from λ_{-1}^0	6000	6000	6000
Target interface	Success probability (attempts per trajectory)		
λ_0^1	0.195 ± 0.008 (20)	0.248 ± 0.01 (20)	0.234 ± 0.01 (20)
λ_1^2	0.294 ± 0.02 (20)	0.330 ± 0.01 (20)	0.313 ± 0.02 (20)
λ_2^3	0.284 ± 0.02 (20)	0.323 ± 0.01 (20)	0.433 ± 0.03 (20)
λ_3^4	0.142 ± 0.01 (6)	0.223 ± 0.01 (6)	0.311 ± 0.02 (6)
Total reactive trajectories found	1285	921	971

TABLE S7. Details of Rosenbluth FFS simulations performed to estimate the kinetics of internal displacement at 340.9 K. The top half of the table reports the initial simulations of flux across the λ_{-1}^0 interface. The bottom half contains the simulation data from the later stages: the total number of trajectories initiated at λ_{-1}^0, the success rates of attempts to reach the next interface and the number of attempts per trajectory performed at each interface.

Order parameter Q	Separation d/nm	Set of base pairs A with $E < E_A$	Nearly-formed base pairs n	Set of base pairs B with $E < E_B$	Number of base pairs X from S_{r_0} with $E < 0$	Number of base pairs Y from S_{r_0} with $E < E_Y$
$Q = -2$	$\sim$	$A \in S_{r_0}$	$n = 0$	$B \in S_{r_0}$	$\sim$	$\sim$
$Q = -1$	$\sim$	$A \notin S_{r_0}$ or $n \neq 0$ or $B \notin S_{r_0}$			$X \geq 13 - \lvert r_0 \rvert$	$Y \geq 13 - \lvert r_0 \rvert$
$Q = 0$	$\sim$	$\sim$	$\sim$	$\sim$	$(X < 13 - \lvert r_0 \rvert$ & $Y < 13 - \lvert r_0 \rvert)$ & $(X > 12 - \lvert r_0 \rvert$ or $Y > 11 - \lvert r_0 \rvert)$	
$Q = m$, $1 \leq m < 12 - \lvert r_0 \rvert$	$\sim$	$\sim$	$\sim$	$\sim$	$(X \leq 13 - \lvert r_0 \rvert - m$ & $Y \leq 12 - \lvert r_0 \rvert - m)$ & $(X > 12 - \lvert r_0 \rvert - m$ or $Y > 11 - \lvert r_0 \rvert - m)$	
$Q = 12 - \lvert r_0 \rvert$	$d < 5.11$	$A \notin S_{r'}$ or $n \neq 0^*$ or $B \notin S_{r'}$			$X \leq 1$	$Y = 0$
$Q = 13 - \lvert r_0 \rvert$	$d \geq 5.11$	$\sim$	$\sim$	$\sim$	$\sim$	$\sim$

TABLE S8. Order parameter definitions for direct FFS simulations of dissociation of misaligned duplexes at 300 K. Order parameters were different for different initial states r_0 due to the differing number of base pairs. $14 - |r_0|$ gives the total number of base pairs possible in the initial misaligned register r_0. r' is any *other* possible bound structure. misaligned structures with more than four base pairs are considered as possible structures r', along with the pseudoknots $\{r_1, r_2\} = \{-4, 8\}$, $\{-6, 6\}$, $\{-6, 8\}$, $\{-8, 4\}$ and $\{-8, 6\}$. $n = 0^*$ indicates that nearly formed base pairs are forbidden, except in pseudoknot cases when nearly-formed base pairs from the relevant registers are allowed. The symbol '$\sim$' indicates that no restriction is placed on this generalised coordinate except those that follow implicitly from the other requirements. During simulations, the system was also monitored to check for the formation of alternative bound structures, through the criteria $A \in S_{r'}$ & $n = 0^*$ & $B \in S_{r'}$. The energy scales $E_A = -1.43\,\mathrm{kcal\,mol^{-1}}$, $E_B = -1.79\,\mathrm{kcal\,mol^{-1}}$ and $E_Y = -0.596\,\mathrm{kcal\,mol^{-1}}$ are used here.

Order parameter Q	Separation d/nm	Set of base pairs A with $E < E_A$	Nearly-formed base pairs n	Set of base pairs B with $E < E_B$	Number of base pairs X from S_{r_0} with $E < 0$	Number of base pairs Y from S_{r_0} with $E < E_Y$
$Q = -2$	$\sim$	$A \in S_{r_0}$	$n = 0$	$B \in S_{r_0}$	$\sim$	$\sim$
$Q = -1$	$\sim$	$A \notin S_{r_0}$ or $n \neq 0$ or $B \notin S_{r_0}$			$X \geq 13 - \|r_0\|$	$Y \geq 13 - \|r_0\|$
$Q = 0$	$\sim$	$\sim$	$\sim$	$\sim$	$(X < 13 - \|r_0\|$ & $Y < 13 - \|r_0\|)$ & $(X > 11 - \|r_0\|$ or $Y > 10 - \|r_0\|)$	
$Q = m,\ 1 \leq m < 6 - \frac{1}{2}\|r_0\|$	$\sim$	$\sim$	$\sim$	$\sim$	$(X \leq 13 - \|r_0\| - 2m$ & $Y \leq 12 - \|r_0\| - 2m)$ & $(X > 11 - \|r_0\| - 2m$ or $Y > 10 - \|r_0\| - 2m)$	
$Q = 6 - \frac{1}{2}\|r_0\|$	$d < 5.11$	$A \notin S_{r'}$ or $n \neq 0$ or $B \notin S_{r'}$			$X \leq 1$	$Y = 0$
$Q = 7 - \frac{1}{2}\|r_0\|$	$d \geq 5.11$	$\sim$	$\sim$	$\sim$	$\sim$	$\sim$

TABLE S9. Order parameter definitions for direct FFS simulations of melting at 340.9 K. Order parameters were different for different initial configurations, due to the differing number of base pairs. $14 - |r_0|$ gives the total number of base pairs possible in a misaligned register r_0, with r_0 being the initial register of the system. r' is any *other* possible bound structure. misaligned structures with at least eight base pairs are considered as possible structures r', and pseudoknots are not included.The symbol '$\sim$' indicates that no restriction is placed on this generalised coordinate except those that follow implicitly from the other requirements. During simulations, the system was also monitored to check for the formation of alternative bound structures, through the criteria $A \in S_{r'}$ & $n = 0$ & $B \in S_{r'}$. The energy scales $E_A = -1.43\,\mathrm{kcal\,mol^{-1}}$, $E_B = -1.79\,\mathrm{kcal\,mol^{-1}}$ and $E_Y = -0.596\,\mathrm{kcal\,mol^{-1}}$ are used here.

	Initial register			
	10	-10	8	-8
Simulations run for flux across λ_{-1}^{0}	2	8	10	9
Initialisation time per simulation /ns	8.5	8.5	8.5	8.5
Crossings of λ_{-1}^{0} (time taken /μs)	39453 (5.3)	79915 (9.0)	99442 (9.4)	90096 (8.4)
Flux across λ_{-1}^{0} /ns^{-1}	7.33*	8.78 ± 0.28	10.6 ± 0.1	10.6 ± 0.1
Target interface	Total attempts/successes at later stages			
λ_0^1	20000 / 856	50000 / 2107	130000 / 6745	140000 / 7509
λ_1^2	40000 / 1497	90000 / 1807	150000 / 10253	150000 / 10016
λ_2^3	3000 / 374	10000 / 2285	50000 / 3484	50000 / 3437
λ_3^4	N/A	N/A	20000 / 3689	20000 / 4336
λ_4^5	N/A	N/A	12488 / 230	7000 / 111

TABLE S10. Simulation results for direct FFS simulations of melting of misaligned duplexes at 300.0 K. The top half of the table describes the initial flux simulations, and the bottom half contains the data from subsequent interfaces. The number of reactive pathways in direct FFS is simply the number of successes at the final interface. *Only two initial calculations of flux were run for this state, making the calculation of errors unreliable. Note that, particularly for the registers ± 10, internal displacement processes were also observed during simulations. These processes are not well described by the FFS order parameter of melting, meaning that they are not accurately sampled in these simulations. An uneven presence of these displacement trajectories for registers ± 10 causes the large differences between individual entries for the two registers in the table. The overall rate of melting (Table S16), however, is similar for the two cases, as it should be. This also suggests that the error on the register 10 estimate is reasonably small.

	Initial register		
	6	4	2
Simulations run for flux across λ^0_{-1}	15	48	20
Initialisation time per simulation /ns	8.5	8.5	8.5
Crossings of λ^0_{-1} (time taken /μs)	149747 (7.7)	479021 (23)	401195 (18)
Flux across λ^0_{-1} /ns^{-1}	19.4 ± 0.4	20.3 ± 0.2	22.2 ± 0.2
Target interface	Total attempts/successes at later stages		
λ^1_0	160000 / 11789	260000 / 18607	250000 / 18150
λ^2_1	23000 / 10425	245000 / 10791	250000 / 11634
λ^3_2	122500/12577	33750 / 5174	32783 / 4795
λ^4_3	5000 / 2717	4500 / 587	24000 / 3963
λ^5_4	N/A	10000 / 3417	20500 / 4618
λ^6_5	N/A	N/A	9000 / 1399

TABLE S11. Simulation results for direct FFS simulations of melting of misaligned duplexes at 340.9 K. The top half of the table shows how many independent initial flux simulations were performed, and the initialisation time for each of these simulations before data was recorded. The bottom half contains the simulation data: the total crossings of λ^0_{-1} and the time simulated in the first stage, and the total attempts and successes of the later stages. The number of reactive pathways in direct FFS is simply the number of successes at the final interface.

Number of base pairs	0	1	2	3	4	5	6	7	8	9	10	11	12	13	14
Biasing weight W at 300 K	0	10^{14}	5×10^{12}	2×10^{11}	10^{10}	5×10^{8}	2×10^{7}	10^{6}	5×10^{4}	2000	100	5	0.2	0.01	5×10^{-4}
Biasing weight W at 340.9 K	0	3×10^{5}	10^{5}	3×10^{4}	10^{4}	3000	1000	300	100	30	10	3	1	1	1

TABLE S12. Umbrella potential used to bias equilibrium simulations of the duplex state. A base pair counts as formed if it has an energy $E < -0.596\,\mathrm{kcal\,mol^{-1}}$.

aligned structures have a dissociation rate that is negligible with respect to internal rearrangement. Simulations were monitored to check for rearrangement into alternative metastable states: trajectories that resulted in internal displacement were ended and counted as 'failures' for the purposes of measuring the dissociation flux.

At 340.9 K, where dissociation is much faster, it was studied for registers $r_1 = 2$, 4 and 6. Registers −2, −4 and −6 should be related to 2, 4 and 6 by the symmetry of the model, and were not simulated for the sake of efficiency. The other results presented here show no significant asymmetry between positive and negative registers, justifying this approach. Further details of the simulations are given in Tables S10 and S11.

E. Characterisation of the equilibrium ensemble

To understand the kinetic results, it is helpful to characterise the equilibrium ensemble of duplex states. VMMC simulations were performed on a pre-formed 14-base-pair duplex at 300 K and 340.9 K, with umbrella sampling used to enhance the sampling of states with a low degree of base-pairing (but forbid full detachment). The bias applied is detailed in Table S12. Four simulations were run for 10^9 VMMC steps at each temperature, with an initialisation period of 10^6 steps. For simplicity, simulations were performed on systems in which only native base-pairing was permitted. During these simulations, the properties of states with base-pairing energies consistent with the penultimate FFS interface of association were saved. The possible states are given below. Following the notation of Table S1, let $E_A = -1.43\,\mathrm{kcal\,mol^{-1}}$ and $E_B = -1.79\,\mathrm{kcal\,mol^{-1}}$. The two classes of states are:

1. One base pair with $E < E_B$ and one other base pair with $E \approx E_A$, with no other base pairs with $E_B < E < E_A$.

2. One or more base pairs with $E_B < E < E_A$ and one other base pair with $E \approx E_B$, with no other base pairs with $E < E_B$.

In practice, to sample these states, $E \approx E_X$ was taken as $E = E_X \pm 0.03\,\mathrm{kcal\,mol^{-1}}$. The equilibrium probability $P(n)$ of n base pairs with an energy of $E < -0.596\,\mathrm{kcal\,mol^{-1}}$ being present was also measured.

We also compared the intrastrand enthalpy (primarily arising from nearest-neighbour stacking interactions) in the single-stranded ensemble at 300 K to the value obtained from averaging over configurations at the penultimate FFS interface of association. The single-stranded ensemble was sampled in an identical fashion to the duplex simulations above, except that the umbrella potential was set to unity in the absence of base pairs, and zero if any base pairs were present. All states were used to calculate the average intrastrand enthalpy.

Allowed base pairs	T/K	flux / $\mathrm{s^{-1}}$	2 bp success probability
Any	300.0	$(7.67 \pm 0.75) \times 10^4$	0.33 ± 0.018
Any	312.5	$(5.68 \pm 0.77) \times 10^4$	0.27 ± 0.028
Any	326.1	$(3.06 \pm 0.43) \times 10^4$	0.18 ± 0.016
Any	340.9	$(1.34 \pm 0.24) \times 10^4$	0.078 ± 0.010
Native only	300	$(6.23 \pm 0.47) \times 10^4$	0.65 ± 0.023

TABLE S13. Total flux from unbound to fully bound state for non-repetitive sequences. Also shown is the probability of successful completion of duplex formation once the system has reached the penultimate FFS interface.

F. Sequence-dependence of association rate

Pairs of 8-base strands were simulated using the Brownian thermostat in a periodic cell of volume 2.09×10^{-21} l, at a temperature 298.15 K (the temperature used in the experiment of Zhang and Winfree[16]). For each sequence, the time taken for association into the full duplex structure was measured 1000 times. In each case, the system was initialised in the same single-stranded configuration, but with distinct nucleotide velocities. Any correlation resulting from using the same configuration is minimal, as the shortest association time in the simulations is three orders of magnitude larger than the equilibration and diffusion time scales in the single-stranded state. As discussed in Appendix S2 D, the time spent in structures with interactions present between the two strands was not included in this estimate of the association time. Errors in the estimates of rates were calculated using the standard error on the mean of 20 independent estimates, each obtained from 50 events. Additional simulations were performed in which base pairing interactions were restricted to native contacts.

S4. RESULTS

A. Hybridisation of non-repetitive sequences

The results of hybridisation simulations for non-repetitive sequences are enumerated in Table S13 (these results are shown in graphical form in the main text, Fig. 2f). We note that the absolute rate at $T = 300$ K ($7.67 \times 10^4\,\mathrm{s^{-1}}$) would, given the concentrations used in the simulations, translate into a bimolecular association rate of $k_{\mathrm{bi}} = 7.71 \times 10^8\,\mathrm{M^{-1}\,s^{-1}}$. This value is approximately 100 times larger than typical experimental measurements.

As discussed in Appendix S2, we expect coarse-grained models to provide faster dynamics than real systems. To speed up simulations we used a diffusion coefficient that is 16 times higher than the experimentally measured one, which accounts for much of the difference. The frequency of initial contacts (as a function of location within the

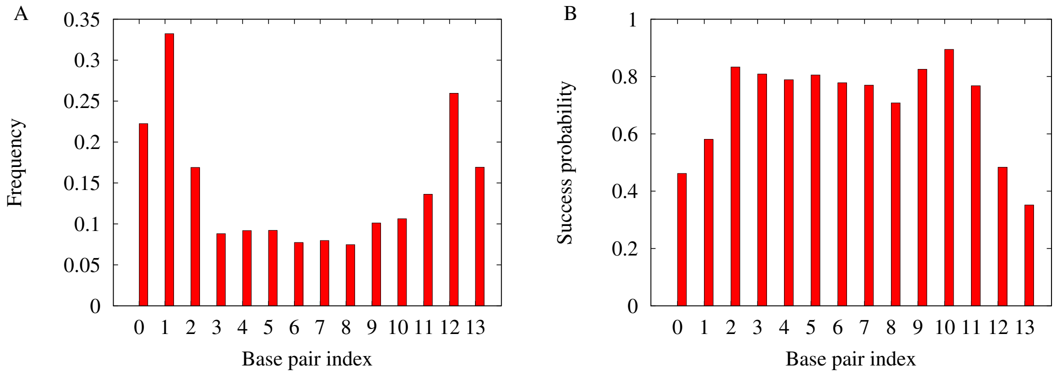

FIG. S4. Binding pathways for non-repetitive sequences. A) Frequency with which a certain base pair appears in states drawn from the penultimate interface of FFS simulations, for a sequence with native-only reactions at 300 K. Base pairs with indexes 0 and 14 are at either end of the duplex. B) Probability of successful duplex formation given the formation of a base pair at the penultimate FFS interface, as a function of base pair index.

Ensemble	Interstrand enthalpy/kcal mol^{-1}	Av. base pair separation /nm
Binding	-7.04	2.84
Duplex	-10.2	2.10

TABLE S14. Properties of ensembles observed during binding and at equilibrium. The binding ensemble consists of states obtained from kinetic simulations at the penultimate FFS interface (as explained in the text), the duplex ensemble contains states that satisfy the same base-pairing criteria, but drawn from an equilibrium sampling of the duplex state.

Register	Flux / s^{-1} 300 K	340.9 K
Correct bonding		
0	$(5.61 \pm 0.49) \times 10^4$	1.15×10^4
misaligned bonding		
2	$(4.99 \pm 0.39) \times 10^4$	1.51×10^4
-2	$(3.68 \pm 0.38) \times 10^4$	1.24×10^4
4	$(3.94 \pm 0.44) \times 10^4$	8.32×10^3
-4	$(3.96 \pm 0.41) \times 10^4$	1.35×10^4
6	$(3.58 \pm 0.45) \times 10^4$	6.41×10^3
-6	$(3.17 \pm 0.40) \times 10^4$	8.64×10^3
8	$(3.02 \pm 0.32) \times 10^4$	~
-8	$(2.68 \pm 0.35) \times 10^4$	~
10	$(3.27 \pm 0.46) \times 10^4$	~
-10	$(2.34 \pm 0.23) \times 10^4$	~
Pseudoknot bonding		
-4,8	173	~
-6,6	801	~
-6,8	308	~
-8,4	14.6	~
-8,6	7.29	~
-8,8		~

TABLE S15. Total flux from unbound to bound states of various alignments for a repetitive sequence. We interpret these fluxes as rates of instantaneous reactions. Pseudoknots rarely form before any complete register: due to this rarity, relative errors for the pseudoknotted results are approximately 100%.

strand) and the probability of successful duplex formation given those contacts are plotted in Figs. S4 A and S4 B. These histograms show that initial contacts are more likely to occur at the ends of the strands, but more likely to succeed if they occur in the centre. However, all initial attachments occur in the transition pathway ensemble with a reasonable frequency.

B. Characterisation of the ensemble of transition pathways, the equilibrium duplex ensemble and the equilibrium single-stranded ensemble

Probabilities of successfully forming the full duplex when hybridisation trajectories reach a state at the penultimate FFS interface are reported in Table S13. These configurations have two base pairs present with a significant interaction strength – a precise definition is given in Section . In the main text, we argue that these transition states are not representative of equilibrium states with the same degree of base pairing. To establish this, we compare states from the penultimate interface of FFS simulations of association in which only native base-pairs were permitted with states obtained from the equilibrium ensemble that satisfy the same base-pairing criteria. For the two ensembles, the average separation of native base-pair contacts and the average overall interstrand enthalpy were measured. The results are given in Table S14.

The difference in interstrand enthalpies between the two ensembles is primarily due to stronger stabilising cross-stacking interactions in the equilibrium configurations. It is not this difference in enthalpy itself, however, that explains our results. In the FFS simulations of dissociation of register -10 at 300 K, for simulations launched from the penultimate interface prior to separation (λ_1^2) full dissociation was observed in 2237 trajectories and

result	Initial register 2	-2	4	-4	6	-6	8	-8	10	-10
0	1.85×10^4	2.68×10^4	1.55×10^3	298	128	41.4	1.04×10^4	5.04×10^3	4.74×10^5	5.30×10^5
2	~		4.95×10^4		867		1.15×10^3	9.51×10^4	2.59×10^5	3.05×10^5
-2	34.0	~		4.54×10^4	30.9	957	1.01×10^5	3.95×10^3	3.33×10^5	3.23×10^5
4	299		~		8.23×10^4	1.43×10^3	2.85×10^3	2.82×10^3	2.88×10^4	4.81×10^5
-4		2.16		~	2.96×10^3	8.89×10^4	1.91×10^3	4.42×10^3	2.81×10^5	3.60×10^4
6			45.9		~	5.46	1.31×10^5	1.38×10^3	1.55×10^4	1.76×10^5
-6				212		~		1.55×10^5	2.69×10^5	2.60×10^4
8					93.7		~		5.49×10^5	1.00×10^5
-8						586		~	8.10×10^4	6.54×10^5
10							409	183	~	1.99×10^4
-10								211	9.98×10^3	~
-4,8							9.91×10^4		2.40×10^4	1.87×10^3
-6,6					1.72×10^3	1.96×10^4	462	1.60×10^3		5.92×10^3
-6,8						1.28×10^3	1.52×10^5		2.89×10^4	2.85×10^3
-8,4					11.7	84.3		5.58×10^4	1.21×10^4	1.52×10^4
-8,6					1.64×10^4			1.80×10^5		1.04×10^4
-8,8							9.84×10^4	8.81×10^4	1.42×10^4	7.65×10^3
melt	~	~	~	~	~	~	8.92×10^3	9.04×10^3	1.47×10^6	1.71×10^6

TABLE S16. Total flux from misaligned states to other (meta)stable states for the repetitive sequence at 300 K. Blank spaces indicates events that could potentially have occurred during simulations, but were not observed. '~' indicates transitions that were not sampled. The most common transition for each initial state is highlighted in yellow, other reasonably frequent results are highlighted in green. Standard errors on the estimates for internal displacement are on the order of 15% for the most common results, rising to 100% for less frequently observed traditions. Standard errors on the estimates of melting are in the range 10 – 25%.

Register	Flux / s^{-1}
Correct bonding	
0	$(1.15 \pm 0.19) \times 10^4$
misaligned bonding	
2	$(1.51 \pm 0.35) \times 10^4$
-2	$(1.24 \pm 0.24) \times 10^4$
4	$(8.32 \pm 1.4) \times 10^3$
-4	$(1.35 \pm 0.33) \times 10^4$
6	$(6.41 \pm 2.8) \times 10^3$
-6	$(8.64 \pm 1.5) \times 10^3$

TABLE S17. Total flux from unbound to various misaligned states for the repetitive sequence at 340.9 K. We interpret these fluxes as rates of instantaneous reactions.

reformation of the full duplex in 5173, despite an average interstrand enthalpy of only $-1.28\,\mathrm{kcal\,mol^{-1}}$ at this interface. Note that many trajectories launched from λ_1^2 reached alternative metastable states, rather than dissociating – in the figures given above we only consider trajectories launched from configurations at λ_1^2 in which no other register of bonding is present, explaining the difference between the numbers quoted and those in Table S10. Rather, the weaker interactions and greater distance between hydrogen-bonding sites in the kinetic ensemble indicate that the geometry of the two strands is not generally conducive to full hybridisation, and not reflective of

	2	4	6
0	1.66×10^5	2.37×10^4	1.37×10^4
2	~	4.54×10^5	1.27×10^5
-2	2.39×10^3	4.54×10^3	1.21×10^4
4	4.22×10^4	~	7.73×10^5
-4	763	179	1.27×10^5
6		6.24×10^4	~
-6	84.6	1.42×10^3	5.03×10^4
melt	6.39×10^4	4.40×10^5	3.64×10^6

TABLE S18. Total flux from misaligned states to other (meta)stable states for the repetitive sequence at 340.9 K. Blank spaces indicates events that could potentially have occurred during simulations, but were not observed. '~' indicates transitions that were not sampled. The most common transition for each initial state is highlighted in yellow, other reasonably frequent results are highlighted in green. Standard errors on the estimates for internal displacement are on the order of 10% for the most common results, rising to 100% for less frequently observed traditions. Standard errors on the estimates of melting are approximately 5%.

states with comparable enthalpy in the bound ensemble. As a result, there is a reasonable probability of strands dissociating even after making initial contacts with significant interstrand interactions, and therefore the process

of duplex formation has a non-negligible negative activation enthalpy.

We have also noted a competing contribution to the activation enthalpy by comparing the intrastrand interactions in the equilibrated single-stranded state with those in the ensemble of states from the penultimate FFS interface. For the unbound ensemble, we obtain an average of $-133\,\mathrm{kcal\,mol^{-1}}$, compared to $-129\,\mathrm{kcal\,mol^{-1}}$ from the states at the penultimate interface of FFS simulations. In the absence of intrastrand base-pairing, this difference is attributable to less effective stacking of the individual strands in the hybridisation ensemble. Disrupting stacking makes it easier for the strands to be in contact without being fully bound, and also optimal stacking configurations are not consistent with duplex geometry.[1] The difference in intrastrand enthalpies contributes to the overall activation enthalpy of binding, tending to make it less negative. However, for our model, the effect of disrupting stacking is a smaller contribution than other effects that favour a negative activation enthalpy.

C. Hybridisation of repetitive sequences

The results of the FFS simulations of the initial hybridisation of repetitive sequences at 300 K are given in Table S15. The probability of formation of each register r_1 is approximately proportional to the number of bonds available, $14 - |r_1|$. The results of FFS simulations of rearrangement and dissociation at 300 K are summarised in Table S16. Equivalent results for simulations at 340.9 K are given in Tables S17 and S18.

As is evident, initial misaligned structures with more than four base pairs tend to rearrange into structures with a greater degree of base-pairing at 300 K. Registers $r_1 = \pm 10$, with only four base pairs, have a similar probability of forming a more strongly-bound duplex and dissociating. We were unable to reliably simulate the relaxation of the relatively stable pseudoknot structures $\{r_1, r_2\} = \{-4, 8\}$, $\{-6, 6\}$, $\{-6, 8\}$, $\{-8, 4\}$ and $\{-8, 6\}$. However, given that each register present in these metastable pseudoknots can form at least six base pairs, it seems likely that these structures would eventually relax to the fully-formed duplex. We emphasise that unlike the six pseudoknots listed above, most pseidoknots relax to a single register reasonably quickly.

To estimate the rate of formation of the fully-formed duplex, we therefore sum over the rate of formation of all structures from the initial simulations, with the exception of registers $r_1 = \pm 10$. In these cases we take the fraction of structures that rearrange into another structure with a higher degree of base-pairing as the fraction that eventually form a full duplex. The result thus obtained is $k_{\mathrm{on}} = 3.8 \times 10^5\,\mathrm{s^{-1}}$, approximately five times larger than the result for non-repetitive sequences given in Table S13. As justified in Appendix S2 D, this analysis ignores the time spent in the metastable intermediates.

At 340.9 K, dissociation is a non-negligible pathway even for the most stable misbonds, $r_1 = \pm 2$. To analyze this case, we considered only the two most likely routes out of each metastable state, those highlighted in green and yellow in Table S18. Calculation of the overall transition rate into the $r_1 = 0$ state is then a relatively simple problem, yielding $k_{\mathrm{on}} = 4.0 \times 10^4\,\mathrm{s^{-1}}$, almost 10 times smaller than for the same sequences at 300 K (this calculation assumes the negative registers behave identically).

S5. DETAILED COMPARISON OF OXDNA WITH 3SPN.1

Here we discuss the differences between our results and those for 3SPN.1, an alternative model of DNA. This discussion is needed because 3SPN.1 has also been used to study hybridisation,[17–21] finding some similar results (such as transitions being complex) but, importantly, finding significantly different pathways towards hybridisation. There are several major differences between oxDNA and 3SPN.1 that are relevant to this analysis.

- Single-stranded DNA in 3SPN.1 consists of unphysically stiff helices,[22] whereas single strands in oxDNA can unstack and hence are more flexible, with a greater degree of conformational freedom. The importance of treating the extra flexibility of ssDNA relative to duplexes is evident in the formation of single-stranded hairpins[23] and in the force-extension properties of ssDNA,[24,25] both of which are accurately reproduced by oxDNA.[1,26]

- The base-pairing interaction in oxDNA is strongly modulated by orientation of the nucleotides[1], meaning that the edges of bases must point at each other to form bonds. This reflects the strongly directional nature of hydrogen bonding. 3SPN.1 has several beads for each nucleotide, but all interactions between beads are isotropic. Thus bonding can occur in configurations in which the bases are close to each other, but not in a realistic orientation for hydrogen-bonding. As discussed by Florescu and Joyeux[27], these isotropic interactions can even lead to unphysical stable states for poly(dA)-poly(dT) in which each nucleotide is bound to two others (although Florescu and Joyeux studied an earlier version of the model, 3SPN.0,[28] the hydrogen-bonding geometry is unchanged in 3SPN.1).

- 3SPN.1 contains an attractive interaction between sugar sites that was introduced to mediate the hybridisation reaction.[22] This attraction provides a stabilising contribution to the system when the single strands are in close proximity to each other, but not bound with hydrogen bonds. Sambriski *et al.*[22] justified this term by referring to the tendency of

DNA duplexes to condense in the presence of multivalent ions, but its role in a model parameterised for monovalent ions is unclear.

Next we discuss how these differences play out for the dynamics of hybridisation. Perhaps the most important geometric difference between the two models is the fact that the single strands in 3SPN.1 are stiff and helical. We show that zippering in oxDNA occurs because the single-strands are relatively flexible: double helices form in stages as bases stack onto the end of the growing duplex. The stiffness of the duplex itself is an emergent property, rather than being imprinted at the level of the single strands. By contrast, in 3SPN.1, hybridisation occurs through the association of two fairly stiff helices, for which the most natural pathway is probably the winding referred to in the detailed study by Schmitt and Knotts.[19]

Another way the flexibility of the strands plays an important role involves the mechanism of internal rearrangement. The intermediates of internal displacement, involving bulged or pseudoknotted states, require significant flexibility in the single strands, and hence these processes will be suppressed by the stiff single strands in 3SPN.1.

Instead of using internal displacement, repetitive strands in 3SPN.1 can slither past each other[17,18,20] in a mechanism 'devoid of significant energy barriers'.[20] This ability to 'slither' suggests that a similar sliding mechanism may also explain how initially misaligned nonrepetitive duplexes relax to the native state.[19,21] Slithering is not observed in oxDNA. In order to undergo slithering, the strands must slide relative to each other along the duplex axis. Performing such an operation with oxDNA would be extremely costly: the system would have to move through an intermediate state in which all base pairs were broken but the strands were still held in a double helical orientation, wrapped around each other. Thus reaching the intermediate state involves an enormous enthalpic cost, with little entropic gain to compensate. By contrast, in 3SPN.1 this process, is 'devoid of significant energy barriers' for repetitive sequences with a repeat unit of two bases.[20] Several factors contribute to this difference. Firstly, the isotropic nature of interactions means that hydrogen-bonding need not be fully disrupted during slithering. Secondly, the attraction between sugar sites stabilises a state in which the two strands are wrapped round each other, but not base-paired. Finally, the fact that 3SPN.1 helices are so stiff means that the conformational freedom of single strands is significantly reduced. Therefore the fact that they must remain helical during the slithering process incurs a relatively smaller entropic penalty than in oxDNA, meaning that it is a viable alternative to dissociating. We note that, although internal displacement via inchworm and pseudoknot intermediates can occur in oxDNA, both processes nevertheless involve significant free-energy barriers associated with initiating the displacement.

Any coarse-grained DNA model makes compromises between accuracy and tractability. In fact such models will never simultaneously reproduce all the properties of DNA, a general attribute of effective coarse-grained systems sometimes called "representability problems".[29] OxDNA was specifically designed in order to reproduce hybridisation thermodynamics as well as the mechanical properties of both single and double strands. We argue here that capturing the strand flexibility as well as the orientational dependence of the effective potentials is crucial if one wants to reproducing the gross features of the hybridisation kinetics we focus on in this paper. Our success at quantitatively reproducing relative rates measured for strand displacement systems[16] gives us confidence in our predictions of similar physical phenomena in hybridisation.

3SPN.1 has some advantages over oxDNA. For example, 3SPN.1 explicitly represents the asymmetric grooves in DNA, allowing structural properties that are sensitive to this feature to be modelled. Electrostatic screening effects are also explicitly included, allowing 3SPN.1 to capture the effects of changing salt concentrations, whereas oxDNA is limited to one salt concentration. Encouragingly, both models show that hybridisation can proceed through complex pathways. Nevertheless, we conclude that oxDNA's representation of hybridisation, involving nucleation and zippering of flexible strands to form stiff helices and the possibility of internal displacement, is more likely to represent true features of real DNA. Of course at the end of the day, the true arbiter of all these predictions will be experiment, and it is likely that slithering and internal displacement will give distinguishable predictions as features such as the repeat length of a repetitive sequence are changed.

[1] T. E. Ouldridge, A. A. Louis, and J. P. K. Doye, "Structural, mechanical and thermodynamic properties of a coarse-grained model of DNA," J. Chem. Phys. **134**, 085101 (2011).

[2] T. E. Ouldridge, *Coarse-grained modelling of DNA and DNA nanotechnology*, Ph.D. thesis, University of Oxford (2011 [Published as a book by Springer, Heidelberg, 2012]).

[3] P. Šulc, F. Romano, T. E. Ouldridge, L. Rovigatti, J. P. K. Doye, and A. A. Louis, "Sequence-dependent thermodynamics of a coarse-grained DNA model," J. Chem. Phys. **137**, 135101 (2012).

[4] T. E. Ouldridge, R. L. Hoare, A. A. Louis, J. P. K. Doye, J. Bath, and A. J. Turberfield, "Optimizing DNA nanotechnology through coarse-grained modelling: A two-footed DNA walker," ACS Nano **7**, 2479–2490 (2013).

[5] J. SantaLucia, Jr., "A unified view of polymer, dumbbell, and oligonucleotide DNA nearest-neighbor thermodynamics." Proc. Natl. Acad. Sci. U.S.A **17**, 1460–5 (1998).

[6] T. E. Ouldridge, "Inferring bulk self-assembly properties from simulations of small systems with multiple constituent species and small systems in the grand canonical ensemble," J. Chem. Phys. **137**, 144105 (2012).

[7] R. L. Davidchack, R. Handel, and M. V. Tretyakov, "Langevin thermostat for rigid body dynamics," J. Chem. Phys. **130**, 234101 (2009).

[8] J. Lapham, J. P. Rife, P. B. Moore, and D. M. Crothers, "Measurement of diffusion constants for nucleic acids by nmr," J. Biomol. NMR **10**, 252–262 (1997).

[9] R. J. Allen, P. B. Warren, and P. R. ten Wolde, "Sampling rare switching events in biochemical networks," Phys. Rev. Lett. **94**, 018104 (2005).

[10] R. J. Allen, C. Valeriani, and P. R. ten Wolde, "Forward flux sampling for rare event simulations," J. Phys.: Condens. Matter **21**, 463102 (2009).
[11] J. Russo, P. Tartaglia, and F. Sciortino, "Reversible gels of patchy particles: Role of the valence," J, Chem. Phys. **131**, 014504 (2009).
[12] D. Frenkel and B. Smit, *Understanding Molecular Simulation* (Academic Press Inc. London, 2001).
[13] S. Whitelam and P. L. Geissler, "Avoiding unphysical kinetic traps in Monte Carlo simulations of strongly attractive particles," J. Chem. Phys. **127**, 154101 (2007).
[14] S. Whitelam, E. H. Feng, M. F. Hagan, and P. L. Geissler, "The role of collective motion in examples of coarsening and self-assembly," Soft Matter **5**, 1251–1262 (2009).
[15] G. M. Torrie and J. P. Valleau, "Nonphysical sampling distributions in monte carlo free-energy estimation: Umbrella sampling," J. Comp. Phys. **23**, 187–199 (1977).
[16] D. Zhang and E. Winfree, "Control of DNA strand displacement kinetics using toehold exchange," J. Am. Chem. Soc. **131**, 17303–17314 (2009).
[17] E. J. Sambriski, V. Ortiz, and J. J. de Pablo, "Sequence effects in the melting and renaturation of short DNA oligonucleotides: structure and mechanistic pathways," J.Phys.: Condens. Matter **21**, 034105 (2009).
[18] E. J. Sambriski, D. C. Schwartz, and J. J. de Pablo, "Uncovering pathways in DNA oligonucleotide hybridization via transition state analysis," Proc. Natl. Acad. Sci. U.S.A. **106**, 18125–18130 (2009).
[19] T. J. Schmitt and T. A. Knotts IV, "Thermodynamics of DNA hybridization on surfaces," J. Chem. Phys. **134**, 205105 (2011).
[20] M. J. Hoefert, E. J. Sambriski, and J. J. de Pablo, "Molecular pathways in DNA-DNA hybridization of surface-bound oligonucleotides," Soft Matter **7**, 560–566 (2011).
[21] T. J. Schmitt, B. Rogers, and T. A. Knotts IV, "Exploring the mechanisms of DNA hybridization on a surface," J. Chem. Phys. **138**, 035102 (2013).
[22] E. J. Sambriski, D. C. Schwartz, and J. J. de Pablo, "A mesoscale model of DNA and its renaturation," Biophys. J. **96**, 1675–1690 (2009).
[23] J. SantaLucia, Jr. and D. Hicks, "The thermodynamics of DNA structural motifs," Annu. Rev. Biophys. Biomol. Struct. **33**, 415–440 (2004).
[24] S. B. Smith, Y. Cui, and C. Bustamante, "Overstretching B-DNA: the elastic response of individual double-stranded and single-stranded DNA molecules," Science **271**, 795–799 (1996).
[25] M.-N. Dessinges, B. Maier, Y. Zhang, M. Peliti, D. Bensimon, and V. Croquette, "Stretching single stranded DNA, a model polyelectrolyte," Phys. Rev. Lett. **89**, 248102 (2002).
[26] F. Romano, D. Chakraborty, J. P. K. Doye, T. E. Ouldridge, and A. A. Louis, "Coarse-grained simulations of DNA overstretching," J. Chem. Phys. **138**, 085101 (2013).
[27] A. M. Florescu and M. Joyeux, "Thermal and mechanical denaturation properties of a DNA model with three sites per nucleotide," J. Chem. Phys. **135**, 085105 (2011).
[28] T. A. Knotts, IV, N. Rathore, D. Schwartz, and J. J. de Pablo, "A coarse grain model for DNA," J. Chem. Phys. **126** (2007).
[29] A. A. Louis, "Beware of density dependent pair potentials," J. Phys.: Condens. Matter **14**, 9187 (2002).
